# Cerebrospinal fluid proteomics in recent-onset Narcolepsy type 1 reveals activation of the complement system

**DOI:** 10.3389/fimmu.2023.1108682

**Published:** 2023-04-12

**Authors:** Ikram Ayoub, Yves Dauvilliers, Lucie Barateau, Thaïs Vermeulen, Emmanuelle Mouton-Barbosa, Marlène Marcellin, Anne Gonzalez-de-Peredo, Catharina C. Gross, Abdelhadi Saoudi, Roland Liblau

**Affiliations:** ^1^ Toulouse Institute for Infectious and Inflammatory Diseases (Infinity), University of Toulouse, Centre National de la Recherche Scientifique (CNRS), Institut National de la Santé et De la Recherche Médicale (INSERM), Université Toulouse III - Paul Sabatier (UPS), Toulouse, France; ^2^ National Reference Center for Orphan Diseases, Narcolepsy, Idiopathic Hypersomnia and Kleine-Levin Syndrome, Department of Neurology, Gui-de-Chauliac Hospital, Centre Hospitalier Universitaire (CHU) de Montpellier, and Institute for Neurosciences of Montpellier, Montpellier, France; ^3^ Institut de Pharmacologie et de Biologie Structurale (IPBS), Université de Toulouse, Centre National de la Recherche Scientifique (CNRS), Université Toulouse III - Paul Sabatier (UPS), Toulouse, France; ^4^ Department of Neurology with Institute of Translational Neurology, University and University Hospital Münster, Münster, Germany; ^5^ Department of Immunology, Toulouse University Hospitals, Toulouse, France

**Keywords:** narcolepsy type 1, neuroimmunology, cerebrospinal fluid, proteomics, complement system, coagulation system, immune response

## Abstract

**Introduction:**

Narcolepsy type 1 (NT1) is a rare, chronic and disabling neurological disease causing excessive daytime sleepiness and cataplexy. NT1 is characterized pathologically by an almost complete loss of neurons producing the orexin neuropeptides in the lateral hypothalamus. Genetic and environmental factors strongly suggest the involvement of the immune system in the loss of orexin neurons. The cerebrospinal fluid (CSF), secreted locally and surrounding the central nervous system (CNS), represents an accessible window into CNS pathological processes.

**Methods:**

To gain insight into the biological and molecular changes in NT1 patients, we performed a comparative proteomics analysis of the CSF from 21 recent-onset NT1 patients and from two control groups: group 1 with somatoform disorders, and group 2 patients with hypersomnia other than NT1, to control for any potential effect of sleep disturbances on CSF composition. To achieve an optimal proteomic coverage analysis, the twelve most abundant CSF proteins were depleted, and samples were analyzed by nano-flow liquid chromatography tandem mass spectrometry (nano-LC-MS/MS) using the latest generation of hybrid Orbitrap mass spectrometer.

**Results and discussion:**

Our study allowed the identification and quantification of up to 1943 proteins, providing a remarkably deep analysis of the CSF proteome. Interestingly, gene set enrichment analysis indicated that the complement and coagulation systems were enriched and significantly activated in NT1 patients in both cohorts analyzed. Notably, the lectin and alternative complement pathway as well as the downstream lytic membrane attack complex were congruently increased in NT1. Our data suggest that the complement dysregulation in NT1 patients can contribute to immunopathology either by directly promoting tissue damage or as part of local inflammatory responses. We therefore reveal an altered composition of the CSF proteome in NT1 patients, which points to an ongoing inflammatory process contributed, at least in part, by the complement system.

## 1 Introduction

Narcolepsy is an orphan chronic sleep disorder characterized by excessive daytime sleepiness. In the majority of narcolepsy cases, referred to as narcolepsy type 1 (NT1), the alteration of the sleep-wake cycles can be associated with cataplexy, which is sudden loss of muscle tone with a preserved state of consciousness, usually triggered by positive emotions (1). The underlying pathology of NT1 is a selective destruction of neurons producing the orexin neuropeptides, also named hypocretins, in the lateral hypothalamus (2, 3). This neuronal loss is reflected in the low to undetectable levels of orexin in the cerebrospinal fluid (CSF), which serves as a highly specific diagnostic biomarker for NT1. Of note, loss of corticotropin-releasing hormone (CRH)-producing hypothalamic neurons was recently reported in NT1 (4). The mechanisms responsible for the loss of orexin-producing neurons are still not well understood but converging evidence for an autoimmune destruction is provided by the genetic and environmental risk factors. Notably, several polymorphisms in immune-relevant genes are associated with NT1, the strongest being the strikingly tight association with the HLA class II allele HLA-DQB1*06:02 (odds ratio>250, 5). Bacterial and viral infections as well as vaccination with Pandemrix against the 2009 pandemic H1N1 flu virus are also associated with increased risk of NT1 (6–8). Supporting the autoimmune hypothesis, recent studies revealed the presence of autoreactive CD4^+^ and CD8^+^ T cells in the blood and CSF of NT1 patients (9–12). However, autoreactive CD4^+^ T cells were also present in HLA-DQB1*06:02-positive healthy controls (10). Furthermore, most orexin-specific CD4^+^ T cells were not restricted to HLA-DQ06:02 (9). One could hypothesize that these T cells recognizing self-antigens expressed in orexinergic neurons migrate into the hypothalamus and contribute to the destruction of orexinergic neurons (13). Indeed, animal models indicate that autoreactive T cells are sufficient to elicit a narcolepsy-like phenotype (14, 15). These studies represent substantial evidence in favor of the immune-mediated loss of orexinergic neurons. In absence of post-mortem brain tissue analysis close to disease onset, analysis of the CSF represents an excellent option to investigate the immunological and neurological changes in the central nervous system (CNS) in this pathological context.

The aim of the present study was to gain insight into the biological and molecular changes in NT1 through CSF proteomics. We opted for high-throughput mass spectrometry-based quantitative proteomics since it provides a sensitive approach to evaluate the relative differential protein abundance between groups. We analyzed CSF from 2 separate cohorts of NT1 patients close to disease onset. NT1 patients were compared to two different sets of controls: persons with somatoform disorders (Controls 1) and patients with sleep disorders other than NT1 (Controls 2), with the aim to shed new light on the immunological and neurological changes associated with the NT1 pathologic process, independently of the potential effect hypersomnia could have on the CSF proteome.

## 2 Materials and methods

### 2.1 Study participants and sample collection

The CSF samples were collected by lumbar puncture from 21 NT1 patients and 2 groups of age- and sex-matched controls; 10 symptomatic controls without any traceable neurological disease and routine CSF cellular and biochemical analysis showing normal or modestly altered values (**Table 1**) and 12 patients with other sleep disorders (2 idiopathic hypersomnia patients and 10 with undefined diagnosis) and normal orexin-A CSF levels. CSF samples were obtained and stored at -80°C until analysis. All NT1 patients but one had cataplexy, all exhibited low levels of orexin-A in the CSF (<110 pg/ml) and carried the HLA-DQB1*06:02 allele. The demographic, clinical and paraclinical features of NT1 patients and controls are summarized in **Table 1** and the overall study design is depicted in **Supplementary Figure 1**. For the clinical correlations, the Narcolepsy Severity Scale (NSS) was used to assess disease severity (16, 17). The NSS has 7 items on excessive daytime sleepiness, 3 items on cataplexy, 2 items on hallucinations, 2 items on sleep paralysis, and 1 item on disrupted nocturnal sleep. The 6 items that assess symptoms frequency are rated with a 6-point Likert scale, while the 9 items that describe the symptom effect on daily life are rated with a 4-point scale. Their sum gives a total score that ranges from 0 to 57 (with the highest scores corresponding to more severe disease).

**Table 1:** Clinical and paraclinical characteristics of NT1 patients and controls included in the study.

	Patients with narcolepsy type 1		Controls	
	Cohort 1 (n=10)	Cohort 2 (n=11)	Subjects with somatoform disorders (n=10)	Patients with other sleep disorders (n=12)
**Mean of age (Years ± SEM)**	24.5 ± 5.11	25.19 ± 3.48	27.8 ± 3.6	26.85 ± 3.44
**Sex (Female/Male)**	3/7	3/8	3/7	9/3
**Time from NT1 onset (Years)**	4.24 ± 1.22	2.85 ± 0.95	—–	—–
**Cataplexy**	10/10	10/11	0/10	0/12
HLA-DQB1*06:02 positive	10/10	11/11	NA	3/12
**CSF Orexin levels < 110pg/ml**	10/10	11/11	NA	0/12

NA, not available.

### 2.2 Sample preparation for label-free proteomics analysis

The CSF samples were thawed on ice and proteins were concentrated by ultrafiltration (Amicon Ultra 0.5mL- 10kDa cutoff, Millipore) at 4°C. For each sample, a depletion of 12 high abundant proteins (α1-Acid Glycoprotein, α1-Antitrypsin, α2-Macroglobulin, Albumin, Apolipoprotein A-I, Apolipoprotein A-II, Fibrinogen, Haptoglobin, IgA, IgG, IgM, Transferrin) was performed using single-use spin columns containing immobilized antibodies (Pierce™ Top 12 Abundant Protein Depletion Spin Columns, Thermo Fisher Scientific). The aim of this depletion step was to reduce the dynamic range of protein concentration and improve the identification and quantification of low-abundant proteins by mass spectrometry (18, 19). Protein samples were lyophilized, resuspended in 50mM ammonium bicarbonate containing 5% of SDS, and then processed for trypsin digestion on S-trap Micro devices (Protifi) according to the manufacturer’s protocol.

### 2.3 Nano-LC-MS/MS analysis of proteins

Peptides were analyzed by nanoscale liquid chromatography coupled to tandem mass spectrometry (nanoLC-MS/MS) using an UltiMate 3000 RSLCnano system coupled to a Q-Exactive-HFX mass spectrometer (Thermo Fisher Scientific, Bremen, Germany). Five µL (i.e., approximately 5µg of peptides) of each sample were loaded onto C-18 precolumn (300 µm ID x 5 mm, ThermoFisher) in a solvent containing 5% acetonitrile and 0.05% trifluoroacetic acid (TFA) and at a flow rate of 20 µL/min. After 3 min of desalting, the precolumn was switched online with the analytical C-18 column (Thermo Scientific Acclaim PepMap 100 C18 2µm, 0.075 x 50mm) equilibrated in 90% solvent A (5% acetonitrile, 0.2% formic acid) and 10% solvent B (80% acetonitrile, 0.2% formic acid). Peptides were eluted using a 10 to 45% gradient of solvent B over 120 min at a flow rate of 350 nL/min. The Q-Exactive-HFX was operated in a data-dependent acquisition mode with the XCalibur software, i.e. peptide ions eluting from the nanoLC column and ionized in the mass spectrometer were regularly surveyed and subsequently isolated in the instrument to be fragmented and obtain sequencing data. Survey MS scans were acquired in a cyclic manner (approximately every second) in the Orbitrap on the 350-1400 m/z range at a resolution of 60000. At each cycle, 12 different peptide ions per survey scan were selected for sequencing by high-energy C-trap dissociation at a resolution of 15000, using a target value of 1e5 ions for filling the C-trap, and a maximum injection time of 22 ms. Dynamic exclusion was employed within 30 seconds to prevent repetitive selection of the same peptide. Two injections were performed for each sample.

### 2.4 Data processing

Raw MS files were processed with the Mascot 2.7 software for database search and Proline 2.1 for label-free quantitative analysis (20). Data were searched against human sequences of the SwissProt database (release UniProt KB 2020_10; 20,386 entries). Carbamidomethylation of cysteines was set as a fixed modification, whereas oxidation of methionine and protein N-terminal acetylation were set as variable modifications. Specificity of trypsin digestion was set for cleavage after K or R, and two missed trypsin cleavage sites were allowed. The mass tolerance was set to 10 ppm for the precursor and to 20 millimass unit (mmu) in tandem MS mode. Minimum peptide length was set to 7 amino-acids, and identification results were further validated in Proline by the target decoy approach using a reverse database at both a peptide spectrum-match and protein false-discovery rate (FDR) of 1%. Label-free relative quantification was performed with Proline by extraction of peptide MS signals intensity to compute a metric for protein abundance. Cross-assignment of peptide ions peaks was enabled across all samples with a match time window of 1 min, after a first alignment of the analytical runs with a time window of +/- 600s.

### 2.5 Statistical analysis

Statistical analysis was performed using an in-house R script integrated in the Proline software. Raw protein abundance values were normalized across all samples, using as a normalization factor the median of protein abundance ratios calculated between each run and a reference run. Quality control of the datasets obtained for both cohorts was performed before and after this normalization step (**Supplementary Figures 2**, **3**). Protein abundances measured from replicate MS injections were averaged, and a filtering step was performed by keeping, for further statistical analysis, only proteins quantified in at least 50% of the individuals in at least one condition (i.e., NT1 and/or control group) and identified with at least 2 tandem mass spectra sequencing scans across all samples. Missing protein abundances were then imputed with a random value chosen in a simulated Gaussian distribution centered, for each sample, on a low intensity value representing background noise. To compare CSF protein abundance in NT1 patients *vs.* controls, the fold change between the mean abundance of proteins in patients and controls was calculated, and the statistical significance of the difference was estimated using a Limma test followed by a Benjamini–Hochberg (BH) correction to adjust the FDR at 1%. The Mann-Whitney test was used to compare selected proteins between groups.

### 2.6 Bio-informatic analysis

Heatmaps of differentially regulated proteins were generated using the Web tool Heatmapper (http://www.heatmapper.ca, 21). Volcano plots were generated using the Web tool VolcaNoseR (https://huygens.science.uva.nl/VolcaNoseR/, 22), a statistical cut-off was set at P ≤ 0.05 to show differentially regulated proteins. Pathway Analysis was performed using the Gene Set Enrichment Analysis (GSEA) feature of the R package clusterProfiler (23). The enriched pathways were selected based on the significance of Enrichment score (P ≤ 0.05).

## 3 Results

### 3.1 Quantitative proteomics reveals differential abundance of proteins in NT1 patients compared to controls without neurological disease

**Figure 1:**
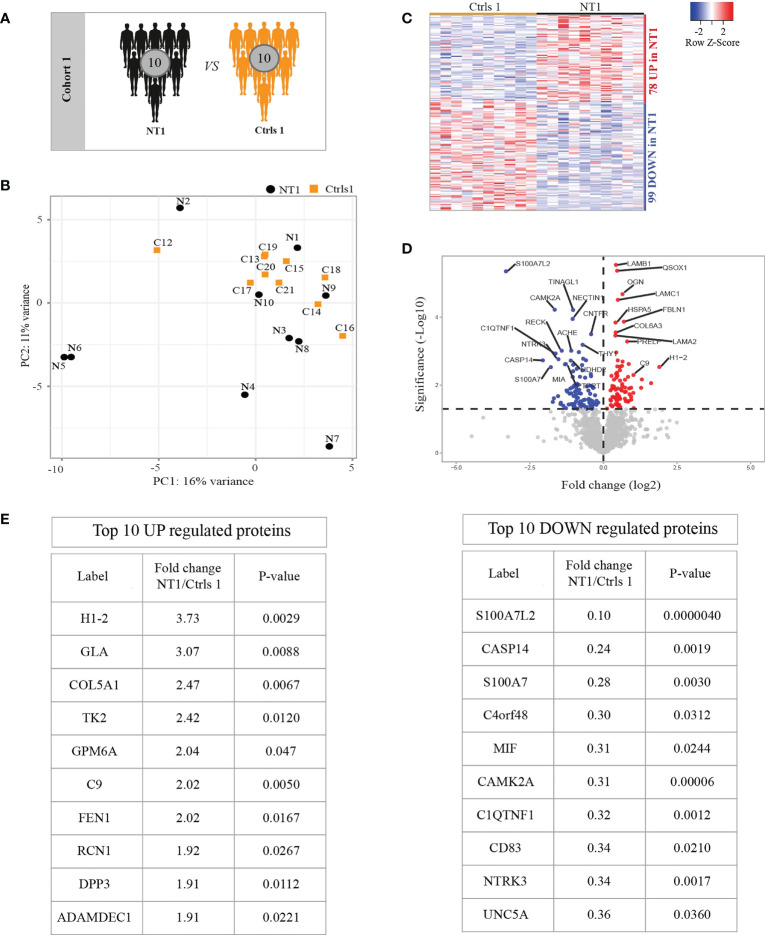
Differentially regulated proteins in the CSF of persons with NT1 compared to controls without neurological disease. **(A)** In cohort 1, the CSF proteome from 10 NT1 patients was compared to that of 10 controls with somatoform diseases. **(B)** Principal Component Analysis (PCA) visualization of the first two principal components. **(C)** Heatmap of proteins with significantly different abundance in the CSF of NT1 patients *vs.* controls (p-value<0.05; Limma test). **(D)** Volcano plot representing the fold change (log2) of each protein in NT1 patients compared to controls and the p-value (-Log10), assessed with the Limma test. A significance threshold was set at p ≤ 0.05 (dashed line). **(E)** The top 10 up-regulated (left) and down-regulated (right) proteins in NT1 patients.

We first analyzed the CSF samples from 10 NT1 patients close to disease onset and 10 symptomatic controls without any traceable neurological diseases, thus we consider these controls the closest to healthy controls (**Table 1** and **Figure 1A**). In order to achieve extensive proteomic coverage, CSF samples were fist depleted from highly abundant proteins, and then analyzed in a non-hypothesis-driven manner, using a fast-sequencing mass spectrometer. A total of 1585 proteins were identified and quantified across all samples, but only proteins identified based on at least 2MS sequencing scans and quantified in more than 50% of a given condition (NT1 or control), i.e., 1397 proteins, were considered for differential analysis. Principal component analysis (PCA) did not show clear segregation of NT1 patients from controls, suggesting the absence of systematic proteomic changes in NT1 patients (**Figure 1B**). Nevertheless, out of the 1397 filtered proteins, 177 proteins were differentially abundant between NT1 patients and controls (p-values ≤ 0.05); 78 and 99 proteins being up-regulated and down-regulated, respectively, in NT1 patients (**Figures 1C, D**). Proteins of immunological relevance, such as Complement factor 9 (C9), ADAMDEC1, DPP3, MIF and CD83 are among the top differentially expressed proteins. Furthermore, the proteins CAMK2A, NTRK3 and UNC5, involved in synaptic plasticity and axon guidance, were significantly down-regulated in NT1 patients, indicating a disturbed neuronal landscape (**Figure 1E**).

### 3.2 Quantitative proteomics of a second cohort reveals differential regulation of proteins unrelated to sleep disorders

**Figure 2:**
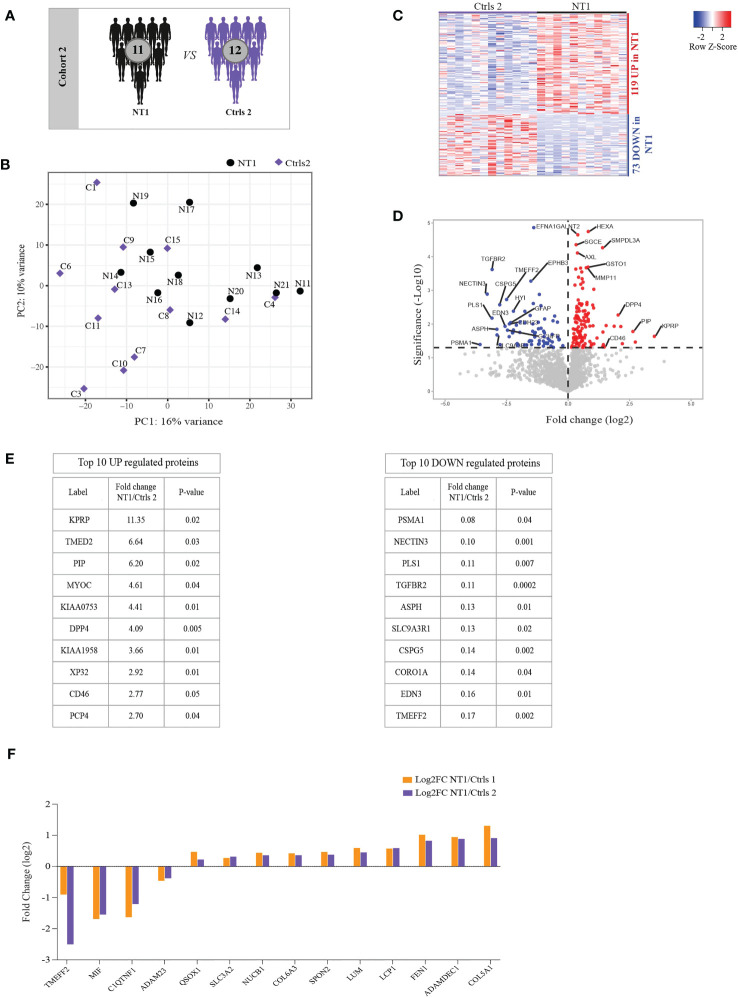
Differentially regulated proteins in the CSF of persons with NT1 compared to patients with other sleep disorders (OSD) and common proteins between cohorts. **(A)** In cohort 2, the proteomic analysis of the CSF from 11 NT1 patients was compared to that of 12 patients with OSD. **(B)** Principal Component Analysis (PCA) visualization of the first two principal components. **(C)** Heatmap of differentially abundant proteins in the CSF of NT1 patients *vs*. OSD (p-value<0.05; Limma test). **(D)** Volcano plot representing the fold change (log2) of each protein in NT1 patients compared to OSD controls and the p value (-Log10), assessed with the Limma test. A significance threshold was set at p ≤ 0.05 (dashed line). **(E)** The top 10 up-regulated (left) and down-regulated (right) proteins in NT1 patients. **(F)** Shared congruently differentially regulated proteins between cohort 1 and cohort 2 and their log2 fold change.

Since sleep architecture disturbances may impact the regulation of inflammatory responses in the CNS (24), we assessed whether the differentially regulated proteins in NT1 are associated with the NT1 disease process or merely the result of sleep alteration. To this end, we used a second cohort including the CSF from 11 NT1 patients close to disease onset and 12 controls with other sleep disorders (OSD, **Figure 2A** and **Table 1**). This proteomics analysis was of a remarkable depth, quantifying a total of 1943 proteins, among which 1626 were filtered for further analysis. PCA did not reveal clear clustering of NT1 patients away from OSD controls (**Figure 2B**). However, 192 proteins were significantly differentially abundant between NT1 and OSD patients; 119 and 73 proteins being up-regulated and down-regulated, respectively, in NT1 patients (**Figures 2C, D**). Although C9 was not significantly elevated in NT1 patients in this second cohort, another protein of the complement system, CD46 (25), was among the top 10 up-regulated proteins in NT1 patients (**Figure 2E**). Abundance of other proteins with immunomodulatory functions such as DPP4 (26), PIP (27) and TGFBR2 (28) was significantly different in NT1 patients. Interestingly, TMEFF2, NECTIN3 and CSPG5 are involved in survival of hippocampal and mesencephalic neurons, synapse formation and function, respectively (29–31). Their significantly decreased expression in the CSF of NT1 patients suggests a potential disturbed synaptogenesis. In order to analyze the signature of NT1 patients independently of hypersomnia or of cohort dependent variations, we searched for differentially regulated proteins shared between the two cohorts. Four and ten proteins were congruently down-regulated and up-regulated, respectively, in NT1 patients (**Figure 2F**). Despite the limited number of shared proteins, the aforementioned immunological and neuronal changes are still observable, as shown in the differential regulation of proteins such as MIF, SLC3A2, SPON2, LUM, LCP1, ADAMDEC1 and the decrease of the synapse related proteins TMEFF2 and ADAM23 in NT1 patients from both cohorts.

**Figure 3:**
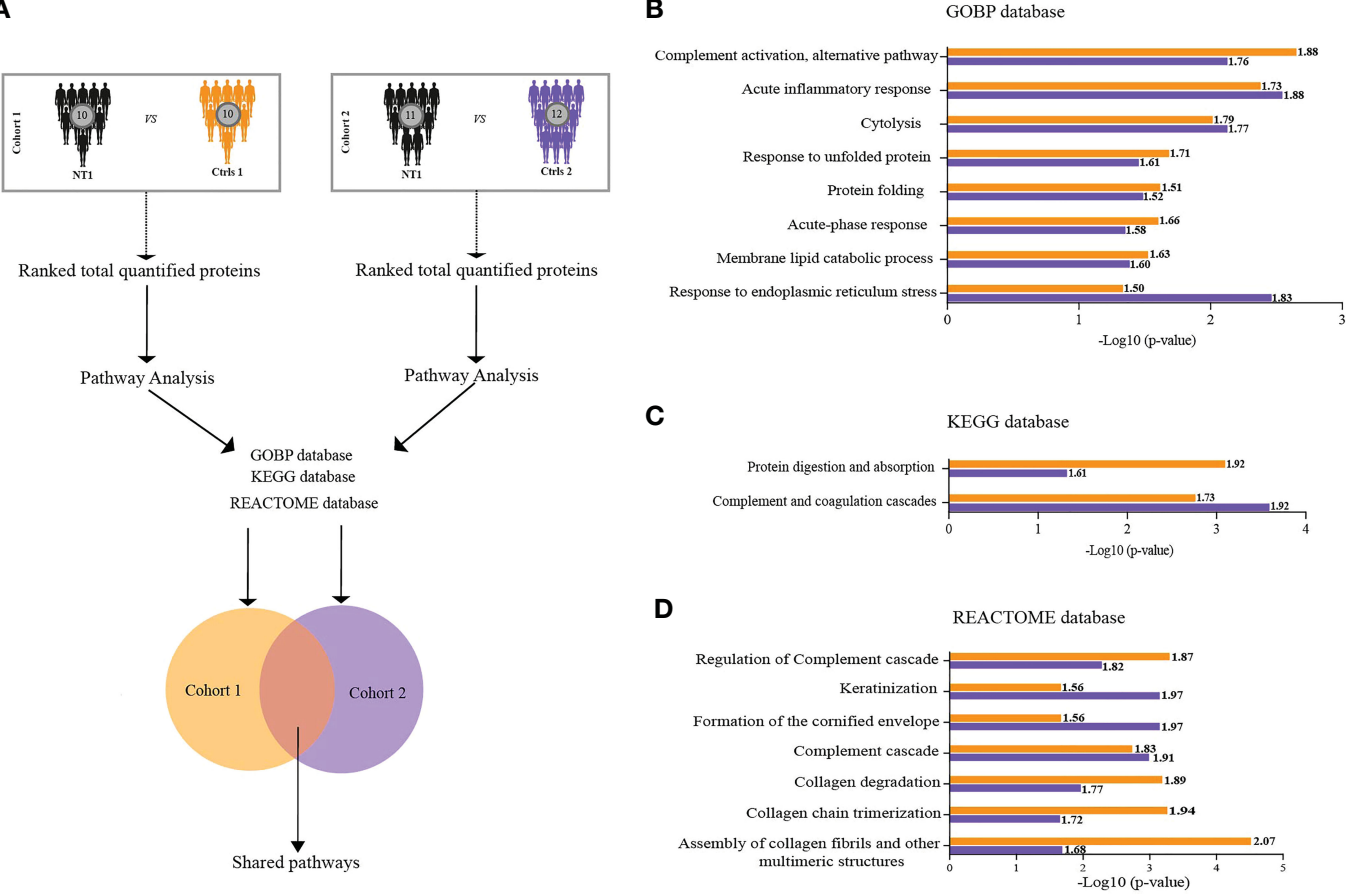
Biological pathways enriched in the CSF of NT1 patients in both cohort 1 and cohort 2. **(A)** Scheme of the analytical design: In each cohort separately, total proteins were ranked based on their fold change then pathway analysis was performed using the GSEA feature of Cluster Profiler. Three databases were interrogated (GOBP, KEGG and REACTOME). Only the statistically significant pathways shared between the two cohorts were considered for further analysis. **(B–D)** Log10 p-values of pathway enrichment in cohort 1 (Orange) and cohort 2 (Purple), in reference to the three databases. Values close to each bar represent the normalized enrichment score (NES), predicting activation or inhibition of a given pathway.

With the aim of understanding the proteomic changes in the CSF of persons with NT1 using a more integrated analytical approach, we evaluated changes at the pathway level. We took advantage of the Gene Set Enrichment Analysis (GSEA) feature provided by clusterProfiler R package and performed pathway analysis in reference to three databases (GOBP, KEGG and REACTOME) in cohort 1 and 2. Shared pathways were then considered for further investigation (**Figure 3A**). All shared pathways present positive normalized enrichment score (NES) values, indicating activation of these pathways in NT1 patients compared to both controls. Interestingly, these results show significant enrichment of pathways related to inflammation in NT1 patients. A concordant activation of the complement system was found using the 3 databases (KEGG, GOBP and Reactome) in NT1 patients from both cohorts. In addition to the inflammatory pathways, a notable activation of pathways such as “response to unfolded protein”, “Membrane lipid catabolic process” and “collagen degradation” was also observed (**Figures 3B–D**).

### 3.3 Increased membrane lipid and collagen degradation in NT1 patients

**Figure 4:**
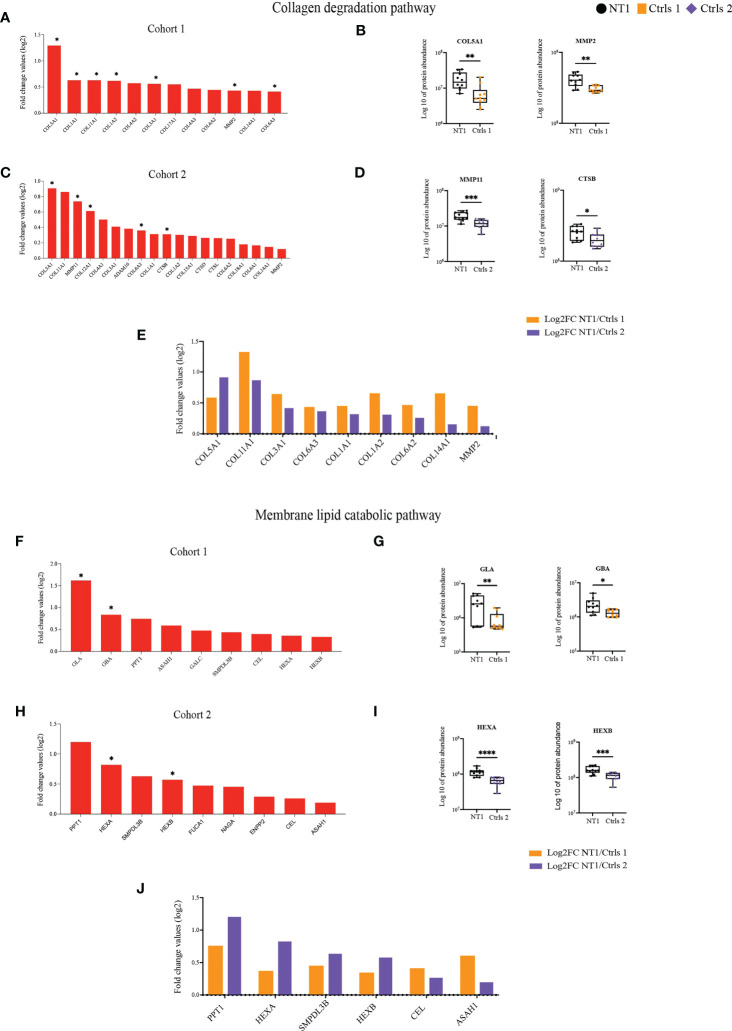
Increased abundance of proteins involved in collagen and lipid degradation in the CSF of NT1 patients. **(A)** The proteins involved in the collagen degradation pathway, their log2 fold change ratio in cohort 1 and **(B)** boxplots of selected proteins. **(C)** The proteins involved in the collagen degradation pathway, their log2 fold change ratio in cohort 2 and **(D)** boxplots of selected proteins. **(E)** Shared collagen degradation proteins between cohort 1 and cohort 2 and their log2 fold change. **(F)** The proteins involved in the membrane lipid catabolic pathway, their log2 fold change ratio in cohort 1 and **(G)** boxplots of selected proteins. **(H)** The proteins involved in the membrane lipid catabolic pathway, their log2 fold change ratio in cohort 2 and **(I)** boxplots of selected proteins. **(J)** Shared membrane lipid catabolic proteins between cohort 1 and cohort 2 and their log2 fold change. P-values in **(A, C, F, H)** are calculated using the Limma test. P-values in **(B, D, G, I)** are calculated with the Mann-Whitney test. *p-value<0.05, **p-value <0.01, ***p-value<0.001, ****p-value<0.0001.

Of particular interest the pathways of lipid and collagen degradation highlight the potential disturbed brain architecture in NT1 patients (**Supplementary Table 1**). Different types of collagens were up-regulated in NT1 patients from cohort 1 and, among them, 7 reached statistical significance, such as COL5A1 (**Figures 4A, B**). Furthermore, the metalloproteinase MMP2, whose main function is to degrade proteins of the extracellular matrix including collagens, was increased in the CSF of NT1 patients (**Figure 4B**). The increased abundance of different types of collagens was also found in NT1 patients from cohort 2 (**Figure 4C**). This increase was statistically significantly for COL5A1, COL12A1 and COL6A3 as well as MMP11 and Cathepsin B (**Figure 4D**). Increased proteins shared between both cohorts are collagens type 1, 3, 5, 6, 11 and 14, as well as MMP2 (**Figure 4E**). The proteins involved in the membrane lipid catabolic process were also up-regulated in NT1 patients from both cohorts (**Figure 4F, H**). However, only GLA, and GBA reached statistical significance in cohort 1, whereas HEXA and HEXAB reached statistical significance in cohort 2 (**Figure 4G, I**). Of note, HEXA and HEXB, the alpha and beta subunits of hexosaminidase, were up-regulated in both cohorts but with a stronger up-regulation in cohort 2 (**Figures 4F, H, J**). In addition, the proteins PPT1, SMPDL3B, CEL and ASAH1 were also shared between both cohorts (**Figure 4J**). Most of these proteins display lysosomal activity and may reflect an intracellular defect in NT1 patients. The enrichment of the pathways “unfolded proteins” and “response to unfolded proteins” in NT1 patients further reinforce this notion. In cohort 1, 20 proteins related to these pathways were up-regulated in NT1 patients, 8 among them reached statistical significance. However, in cohort 2, 5 proteins were associated to these pathways and one protein significantly up-regulated (**Supplementary Figure 4A**). Only the protein HSPA6 was common between both cohorts (**Supplementary Figure 4B**).

Pathways differentially represented only in the comparison between NT1 and OSD patients could also be of interest, as those pathways are detected in NT1 independently of sleep effects on proteome. However, they should be interpreted cautiously due to the absence of significant difference in cohort 1. Among them, the neurotrophin signaling pathway was in particular down-regulated in NT1 patients from cohort 2 (**Supplementary Figure 4C**). Moreover, out of the 16 proteins identified in this pathway, 10 were also similarly down-regulated in NT1 patients from cohort 1 (**Supplementary Figure 4D**). These data suggest a defective neuro-regenerative process in NT1 patients.

### 3.4 Over-activation of the complement and coagulation cascades in NT1 patients

**Figure 5:**
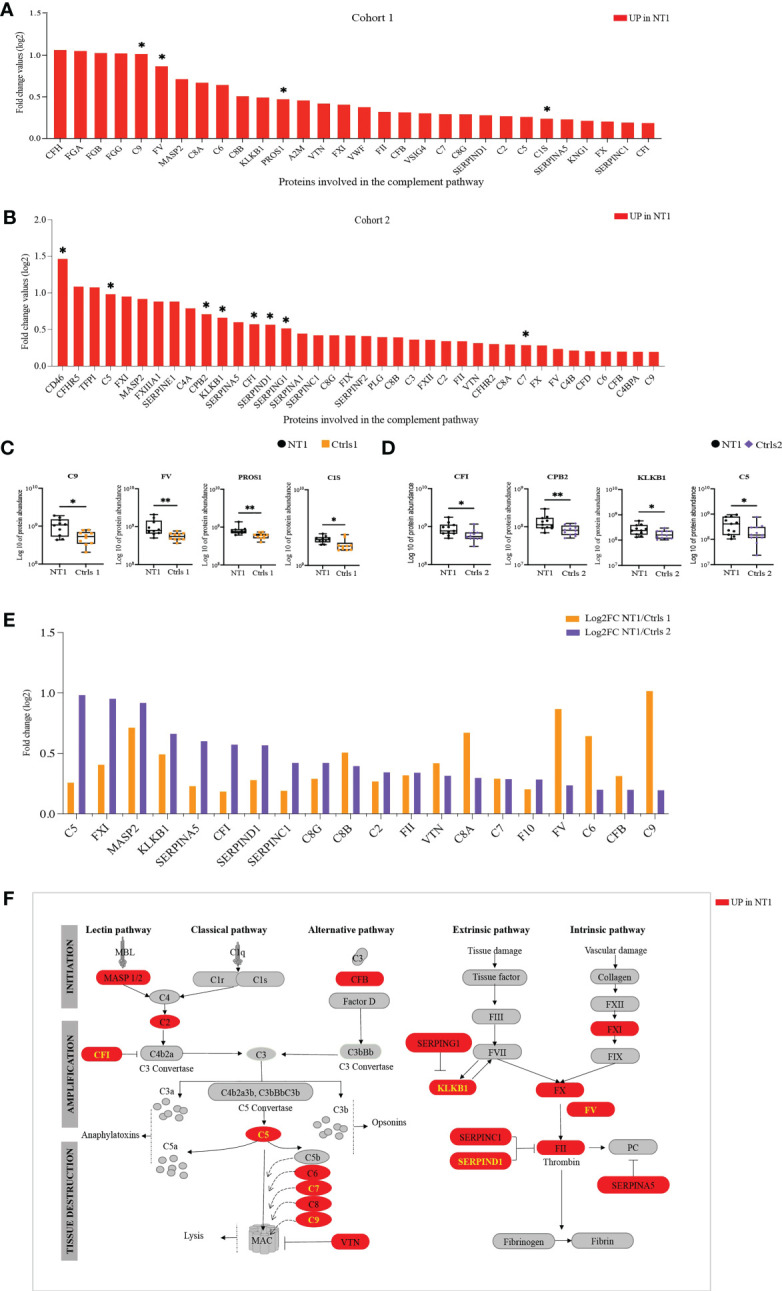
The complement and coagulation pathway are enriched in NT1 patients. **(A)** The proteins involved in the complement and coagulation pathway, their log2 fold change ratio in cohort 1. **(B)** The proteins involved in the complement and coagulation pathway, their log2 fold change ratio in cohort 2. **(A, B)** The proteins that are significantly regulated in the CSF of NT1 patients are indicated with a star (p-value<0.05; Limma test). **(C)** Boxplots of the differentially regulated proteins in cohort 1. **(D)** Boxplots of selected proteins in cohort 2. *p-value<0.05, **p-value <0.01; Mann Whitney test. **(E)** Complement and coagulation proteins differentially regulated in both cohort 1 and cohort 2 and their log2 fold change. **(F)** Representation of the complement and coagulation proteins highlighting in red proteins up-regulated in the CSF of NT1 patients from both cohorts.

Given the congruent deregulation in the complement and coagulation pathways identified in the CSF of NT1 patients in the 3 queried databases, we analyzed in more detail the variation in abundance of all the detected proteins from these pathways. There was a consistent up-regulation of complement and coagulation proteins in the CSF of NT1 patients in both cohorts, with 4 such proteins reaching statistical significance in cohort 1, and 8 in cohort 2 (**Figures 5A, B**). Specifically, C9, FV, PROS1 and C1s were all significantly up-regulated in NT1 patients compared to controls without neurological disorders (**Figure 5C**). In cohort 2, proteins from the complement cascade (C5), the coagulation cascade (KLKB1) and regulators of their activation (CPB2 and CFI) were among the significantly up-regulated proteins (**Figure 5D**). Of particular interest, 20 proteins from the complement and coagulation cascades were up-regulated in both cohorts (**Figure 5E**) and, among them, 7 proteins (CFI, C5, C7, C9, KLKB1, F5, SERPIND1) were significantly up-regulated in one of the cohorts (highlighted in yellow on **Figure 5F**). These included proteins involved in the initiation of the lectin and alternative pathways (C2, MASP2 and CFB), and nearly all the components (C5, C6, C7, C8 and C9) of the membrane attack complex (MAC), which represents the final lytic phase of complement activation. Regulators of complement activation, such as CFI and VTN, were also up-regulated in NT1 patients. Proteins of the extrinsic and intrinsic coagulation cascades (FX, FXI, FV and FII), as well as regulators of the coagulation (KLKB1, SERPINA5, SERPINAC1 and SERPINAD1), were up-regulated. The changes in complement and coagulation cascades observed in the CSF of NT1 patients are integrated in a schematic form (**Figure 5F**).

**Figure 6:**
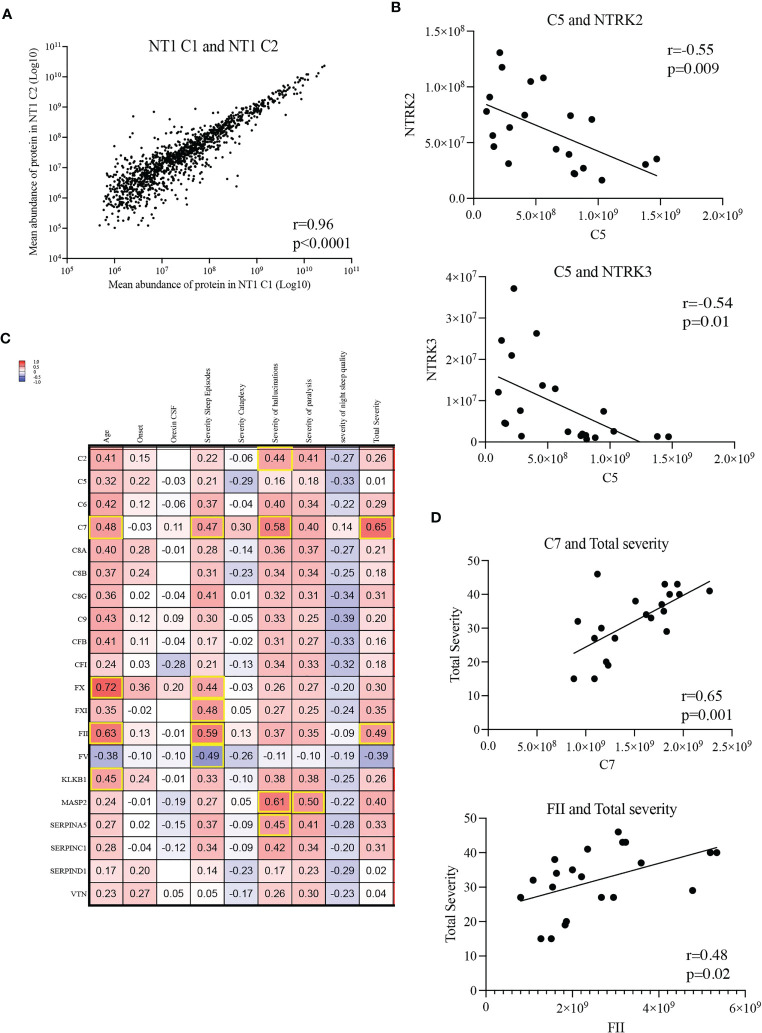
Correlations between complement and coagulation proteins and clinical parameters. **(A)** Correlation of mean abundance of all quantified common proteins shared between NT1 patients of both cohorts. **(B)** Inverse correlation between the abundance of protein C5 with that of NTRK2/TrkB and NTRK3/TrkC in the CSF of the 21 studied NT1 patients. **(C)** Shared proteins of the complement and coagulation pathways correlated with clinical parameters (age, time from onset, orexin CSF levels, severity of sleep episodes, severity of cataplexy, severity of hallucinations, severity of sleep paralysis, severity of night sleep disruption and total disease severity). All NT1 patients from both cohorts were included in this analysis. The values in the heatmap represent the coefficients of correlation assessed with Pearson test and highlighted squares represent statistically significant correlations. **(D)** Correlation of proteins C7 and FII with NT1 total severity.

Under pathological conditions, excessive synaptic pruning mediated by the complement causes loss of synapses (32, 33). Given the important role of neurotrophin signaling in synapse organization, we hypothesized that a decreased level of neurotrophins and their respective receptors could be associated with a loss of synapses. Therefore, we performed correlation analyses between the aforementioned complement and coagulation proteins and the neurotrophin signaling proteins. Since NT1 patients from both cohorts were selected based on the same clinical criteria and that the mean protein abundance correlated strongly between cohorts (**Figure 6A**), including the proteins differentially abundant in NT1 (**Supplementary Figures 5**, **6**), we performed this analysis on the 21 NT1 patients studied. Strikingly, the abundance of several complement proteins, such as C5, are negatively correlated with the abundance of the key neurotrophin receptors NTRK2/TrkB and NTRK3/TrkC (**Figure 6B**; **Supplementary Figure 7**), suggesting a potential neurotoxicity of the complement system in NT1.

Moreover, we performed comparisons between the complement and coagulation proteins and the clinical disease severity metrics. While levels of some complement and coagulation proteins (C2, FX, FXI, FV, MASP2 and SERPINA5) correlated with specific parameters of disease severity (**Figure 6C**), levels of the C7 and FII proteins positively correlated with total disease severity (including severity of sleep episodes, cataplexy, hallucinations, sleep paralysis and night sleep quality) in NT1 patients from both cohorts (**Figure 6D**). To determine if complement and coagulation system activation could be involved in initiating the orexinergic neuron loss, we stratified NT1 patients from both cohorts depending on whether the CSF was harvested close (<2years) or longer (>2years) after disease onset and compared their abundance of the 20 shared complement and coagulation proteins. No increase of complement and coagulation activation was observed in NT1 patients close to disease onset, thereby not supporting an initiating role for this pathway in the disease process (**Supplementary Figure 8**).

## 4 Discussion

In the present study, we compared the CSF proteome of NT1 patients close to disease onset with that of two control groups. The first analysis, involving the CSF from 10 NT1 patients and 10 controls with somatoform manifestations, revealed that proteins of immunological relevance were increased, while proteins related to synaptogenesis were decreased in NT1 patients. However, sleep disturbance associated with NT1 can increase circulating markers of inflammation, such as C-reactive protein (CRP) and IL-6, as demonstrated in a large meta-analysis (34). To rule out this potential bias, we compared CSF proteome from a second cohort of NT1 patients close to disease onset with CSF proteome of patients with OSD.

By comparing the differentially regulated proteins across cohorts, we identified 14 biomarkers (TMEFF2, MIF, C1QTNF1, ADAM23, QSOX1, SLC3A2, NUCB1, COL6A3, SPON2, LUM, LCP1, FEN1, ADAMDEC1, COL5A1) associated with NT1, of potential utility if confirmed in larger cohorts. The main function of each protein is detailed in a **Supplementary Table 2**. Perturbation of abundance of these proteins in persons with NT1 seems to reflect a disturbed extracellular matrix and neuronal function. To our knowledge, no other unbiased proteomics study of the CSF of NT1 patients has been reported. However, dosage of targeted proteins in the CSF has been performed showing an increase in IL-4 (15) and no differences in 51 cytokines and chemokines (35).

To better understand the pathological processes in NT1, we used the GSEA feature of the clusterProfiler tool. This method relies on the analysis of all available data without using an arbitrary cutoff to select differentially regulated proteins, thus allowing for discovery of pathways with subtle yet coordinated changes. Our results show activation of the complement and coagulation system in NT1 patients from both cohorts. The complement system contains over 40 proteins involved in clearance of pathogens and apoptotic cells but also in modulating adaptive immune responses. It can be activated through 3 main pathways: classical (CP), lectin (LP) and alternative (AP). The CP is activated after binding of the C1q to antigen-antibody complexes or other molecules such as β-amyloid, CRP, DNA or apoptotic bodies. The LP is activated through pattern recognition receptors exposed by pathogens or by altered host cells. The AP is dependent on the spontaneous hydrolysis of circulating C3 into C3(H2O) on cellular surfaces. An additional activation pathway has been described that involves the extrinsic pathway of the coagulation system (36).

Levels of some complement proteins in sera of NT1 patients were recently investigated, showing an increase in C1q levels (37). However, to our knowledge, no other study showed the association of complement and coagulation proteins with NT1 in the CSF. Nevertheless, there is growing evidence of complement system activation in neuroinflammatory and neurodegenerative diseases, including brain traumatic injury (38, 39), amyotrophic lateral sclerosis (40, 41), multiple sclerosis (MS, 42, 43), antibody-mediated autoimmune encephalitidies such as MOG antibody (MOGAD) and GAD associated diseases, i.e., neurological disorders associated with anti-glutamic acid decarboxylase autoantibodies such as stiff-person syndrome, cerebellar ataxia and temporal lobe epilepsy (44–46).

Considering all complement proteins identified in our dataset, 22 complement proteins (C9, MASP2, C8a, C6, C8B, VTN, CFB, VSIG4, C7, C8G, C2, C5, C1s, CFI, CD46, CFHR5, C4a, C3, CFHR2, C4b, CFD, C4BPA) out of the 40 complement proteins are consistently up-regulated in NT1 patients. Up-regulation of C1s, MASP2, CFB and CFD points to an activation of the CP, LP and AP, raising the question of whether complement activation is initiated through immune complexes, pathogen recognition or apoptotic debris. Absence of neuronal autoantibodies in the CSF of NT1 patients argues against a role of immune complexes in the NT1 pathogenesis (47). Activation of the complement system through recognition of apoptotic neuronal debris seems a more likely hypothesis. However, searching for antibodies directed against pathogens, such as *influenza* virus, in the CSF of NT1 patients could be of relevance.

Regardless of the initiator, complement activation converges into the mediation of 3 main functions: 1) direct lysis of targeted surfaces through the MAC; 2) recruitment of inflammatory cells through generation of the C5a and C3a; and 3) clearance of targeted surfaces through opsonization with C3b and C4b (36). In the developing brain, the complement is involved in synaptic pruning mediated by microglial cells (48). This physiological process of eliminating unwanted synapses has been shown to be aberrantly activated in aging brain and in neurodegenerative diseases as well as in progressive MS (49–52). In a mouse model of AD, it has been shown that C1q, the initiating protein of the classical complement, is increased and associated with synapses before overt plaque deposition (33). Moreover, in MS patients and mouse models of MS, C1q and C3 have also been associated with synapse loss (32, 43). Although, our study did not show an upregulation of C1q, it is tempting to speculate that activation of the complement system (evidenced by the upregulation of other complement proteins) could lead to activation of an inflammatory type of microglia, which could have a role in initiating or worsening the orexin neuron damage by excessive synaptic pruning. Supporting this hypothesis is the up-regulation of the C3 protein in cohort 2 and the increased of C3b and C3bi receptor (*VSIG4*) in cohort 1. The exact contribution of complement activated microglia in NT1 pathogenesis remains to be explored.

This increase in complement proteins raises the question about their cellular source. The major source of complement proteins is hepatocytes (53) but it is now well established that different CNS resident cell types are capable of synthesizing complement proteins including neurons, astrocytes and microglia (54, 55). The absence of a compromised BBB in NT1 patients suggests a local production by brain cells rather than a leakage of serum derived proteins. Notably, C1q has been identified as a mediator of inflamed microglia in MS tissue and in the mouse model of EAE (43).

Our data show that in the CSF of persons with NT1 the abundance of most proteins of the complement, such as C5, is inversely correlated with that of proteins of the neurotrophin signaling pathway, such as NTRK2 and NTRK3. The interpretation of these data is uncertain. The decrease in neurotrophin signaling could be the result of complement mediated synaptic pruning. However, given the important role of neurotrophin signaling in neuronal survival and repair, this decrease could contribute to the loss of orexinergic neurons, independently of complement activation. It is also possible that the decrease in neurotrophin signaling is a consequence of the absence of orexin peptides, as suggested by the increase in mRNA expression of Neurotrophin 3 (NT-3) and Brain derived neurotrophic factor (BDNF) in primary neuronal cultures, after exposure to orexin peptides (56).

Our CSF proteomics data reveal an up-regulation of the coagulation system in NT1 patients. Activation of this pathway comprises a sequential cascade of proteolytic events leading to the formation of thrombin (FII) and the subsequent conversion of fibrinogen to fibrin. Increasing evidence shows association of abnormal coagulation pathways with neuroinflammatory and neurodegenerative diseases. Components of the coagulation system, increased in NT1 patients, have been strongly linked to the pathogenesis of multiple diseases of the CNS. Fibrinogen has been shown to induce microglia activation as well as recruitment of Th1 myelin-specific cells and Fibrin-targeting immunotherapy inhibited autoimmunity- and amyloid-driven neurotoxicity in animal models of MS and AD, respectively (57, 58). Furthermore, increased levels of thrombin have pathological effects in AD, PD and MS (59).

Our results suggest that the complement and coagulation system do not initiate the pathogenic process of orexin neurons. However, the small number of NT1 patients studied poses the challenge of limited statistical power for NT1 subgroup analysis.

An unexpected observation was the increased abundance of multiple enzymes related to catabolism of sphingolipids in the CSF of NT1 patients. This type of lipids is highly abundant in the brain (60) and the impairment of their metabolism could induce alteration of different metabolic pathways such as autophagosome lysosome pathway, the ubiquitin proteasome system, inflammation, mitochondrial dysfunction and oxidative stress (61). Most of the lipid catabolic enzymes increased in NT1 patients are located in the lysosomal compartment and are involved in catabolism of glycosphingolipids, glucosylceramides, ceramides, galactoceramides, sphingomyelin and gangliosides. Furthermore, loss-of-function mutations in the proteins GLA, GBA, PPT1, ASAH1, GALC, HEXA, HEXB and FUCA1 are associated with lysosomal storage disorders, including Fabry’s disease, Gaucher’s disease, Neuronal ceroids lipofuscinosis, Krabbe’s disease, Sandhoff and Tay Sachs disease (62). In contrast, our results show up-regulation of these proteins, suggesting an increase in lipid metabolites. As the heterodimer protein HEXA/HEXAB has a role in the catabolism of GM2 gangliosides enriched in the CNS, these results may also reflect enhanced degradation of glial and neuronal cell membranes. Interestingly, increased expression of the enzyme GALC was shown in post mortem brains with different neuropathological conditions including AD. Correspondingly, increased levels of ceramide was detected in brains from patients with neurodegenerative disorders (63, 64). Although protein levels in the CSF may not always correlate with those in the brain tissue itself, the CSF is more representative of the changes in the CNS secretome compared to blood (65). Therefore, an alteration in lipid metabolites could be an important feature in the pathogenesis of NT1. Study of CSF lipidomics would be an interesting next step to further characterize the nature of these alterations.

Our results also show that many types of collagens are abundant in the CSF of NT1 patients. Collagens are principally found in connective tissues associated with the nervous system, the basement membranes between the nervous system and other tissues and the sensory end organs (66). In line with our results showing an increase in the collagen IV in NT1 patients (α2 in cohort 1 and α1 in cohort 2), a similar increase in collagen IV has been shown in isolated cerebral micro-vessels (67) in post mortem brains of PD patients and in animal models of PD (68, 69), suggesting an association of neurodegenerative diseases with abnormal basal lamina (70). However, as discussed above, no biochemical evidence of BBB disruption could be detected in CSF analysis of NT1 patients. Possible explanations to this discrepancy could be that in NT1 patients, the BBB breakage is minor and restricted to the lateral hypothalamus where the orexinergic neurons are located, or perhaps an alteration in the basal lamina has other consequences that goes beyond the disruption of BBB and that should be addressed in future studies. Another potential explanation for the increased abundance of collagen proteins in NT1 is the increased abundance of the matrix metalloproteinases (MMP2 and MMP11). In addition to the extracellular matrix proteins including collagens, MMP degrade growth factors, receptors and adhesion molecules (71). MMP2 and MMP9 have been found to be increased in the CSF of patients with MS (72, 73).

One limitation of this study is the low number of participants. However, we selected a homogenous group of proven NT1 patients, with low/undetectable CSF orexin levels and cataplexy, yet close to disease onset. These patients are rare as NT1 is an orphan disease and its diagnosis occurs on average 7 years after disease onset. Therefore, for most NT1 patients the spinal tap is performed late in the disease process at a time-point unlikely to capture ongoing pathological processes. We are fully aware that the CSF samples were nevertheless taken after onset of the biological disease process and that the changes identified in this study could be a consequence, rather than a cause, of the pathogenesis. However, we carefully selected the NT1 patients to mitigate the best we could this possibility. Another limitation is the analysis of the two cohorts in two separate experiments. Nevertheless, a good correlation was observed in the abundance of CSF proteins between the two NT1 cohorts. Moreover, we could identify several proteins and pathways displaying the same pattern of regulation in NT1 patients, compared to two distinct groups of controls. Using these 2 sets of controls allowed for the identification of consistent alterations independent of sleep disturbances.

Identifying whether the activation of the complement system is a consequence of neuronal loss or whether it contributes (as a primary or an amplifying event) to this loss, through recruitment of innate and adaptive immune cells or by directly causing orexinergic neuron damage or synaptic pruning, is an exciting avenue for future research. Follow-up studies of NT1 patients compared to patients with recent neuronal loss, such as minor stroke, could elucidate if complement activation is a cause or a consequence of orexin neuron loss.

In conclusion, our study identified an over activation of the complement and the coagulation system in the CSF of NT1 patients with a correlation between C7 and FII proteins and disease severity. Furthermore, other pathways were altered including the catabolism of lipids and collagens. Although a recent study did not detect an increase in classical neurodegeneration markers in the CSF of NT1 patients (74), our unbiased proteomics study reveals a striking resemblance of pathological mechanisms, to neurodegenerative diseases such as AD and PD. Indeed, the presence of lysosomal defects, responses to unfolded proteins, the seemingly disturbed extracellular matrix, the decreased synaptogenesis and the activation of complement system are all shared features.

## Data availability statement

The data presented in the study are deposited in the ProteomeXchange repository, accession number PXD041093. 

## Ethics statement

The studies involving human participants were reviewed and approved by Comité de Protection des Personnes Nord-Ouest I, CPP 00035/200018. Written informed consent to participate in this study was provided by the participants’ legal guardian/next of kin.

## Author contributions

Study conception and design: RL, YD, IA, and AS. Sample and patient data collection: YD, LB, CG and IA. Experiments and data analyses: IA, EM-B, MM, AG-d-P and TV. Writing original draft: IA and RL. All authors contributed to manuscript revision, read, and approved the submitted version. Funding acquisition: RL and YD.
