# Supplementary material for: Cerebrospinal fluid proteomics in recent-onset Narcolepsy type 1 reveals activation of the complement system
Source: Front Immunol. 2023 Apr 12;14:1108682. doi: 10.3389/fimmu.2023.1108682 (PMC10130643; doi:10.3389/fimmu.2023.1108682)
Supplement: Supplementary file 1 [file DataSheet_1.docx]

Supplementary Material

Article Title

First Author*, Co-Author, Co-Author

*** Correspondence:** Corresponding Author: email@uni.edu

# Supplementary Data

Supplementary Material should be uploaded separately on submission. Please include any supplementary data, figures and/or tables.

Supplementary material is not typeset so please ensure that all information is clearly presented, the appropriate caption is included in the file and not in the manuscript, and that the style conforms to the rest of the article.

# Supplementary Figures and Tables

For more information on Supplementary Material and for details on the different file types accepted, please see [here](https://www.frontiersin.org/guidelines/author-guidelines#supplementary-material).

## Supplementary Figures

**
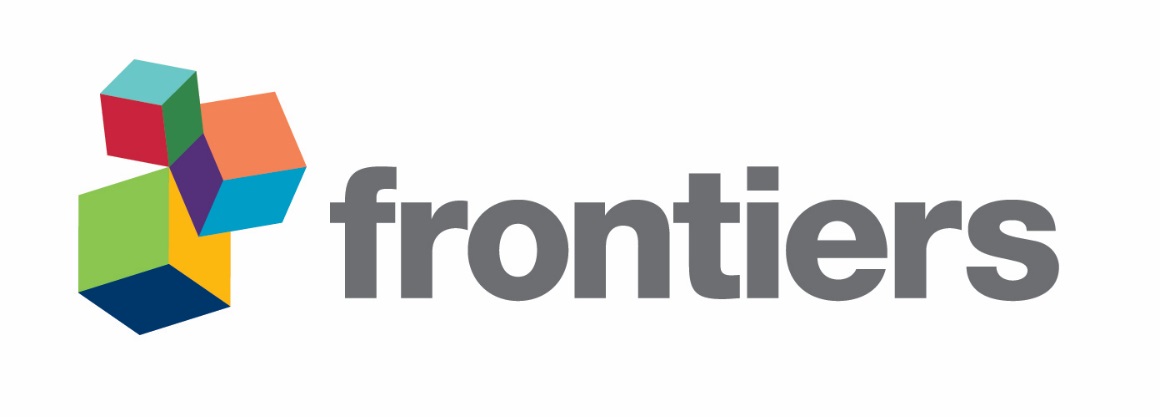
**

**Supplementary Figure 1. Study design and methodology. (A)** The CSF from NT1 patients were analyzed and compared to two different control groups; controls with somatoform clinical manifestations (Ctrls 1) and patients with other sleep disorders (Ctrls 2). **(B)** Following the lumbar puncture, the CSF samples were centrifuged to collect the CSF supernatant. Proteins were then extracted and 12 major proteins were depleted. Proteins were next processed for trypsin digestion. **(C)** Peptides were analyzed by nano-LC-MS/MS in duplicates then identified and quantified using the Proline software. **(D)** Differential analysis was determined by assessing the fold change ratio between NT1 and controls and the statistical significance of difference was assessed using the Limma test. **(E)** Data from both cohorts was integrated using two approaches: (Left) shared differentially regulated proteins between cohorts (p-value <0.05) and (Right) shared pathways between cohorts.

**Supplementary Figure 2**. **Quality control of the mass spectrometry data obtained after analysis of persons with NT1 compared to controls without neurological disease.** **(A**) Distribution of the log-transformed intensity values for all proteins quantified in each LC-MS analysis. Two replicate LC-MS analyses were performed for each of the NT1 patients (n=10) and controls (n=10), resulting in 40 analytical runs. Distributions are shown before (left) and after (right) normalization of the data. **(B)** Pearson correlation coefficients for each pairwise comparison between the 40 analytical runs, showing high correlation between the experiments. (**C**) Number of proteins identified by MS/MS sequencing in all the samples and MS experiments (technical MS replicate runs are plotted side by side), indicating homogeneous level of protein identification across samples. (**D**) Data completeness after assignment of protein signals across all runs. Intensity values of proteins that were not sequenced and identified in some of the runs were retrieved by MS signal extraction using the “match between runs” procedure for label-free quantification. The residual number of missing values after this cross-assignment is indicated for each analysis and is typically around 15%. (**E**) Distribution of the coefficients of variation of intensity values across all biological samples, for the entire population of quantified proteins, showing a mode around 30%.

**Supplementary Figure 3**. **Quality control of the mass spectrometry data obtained after analysis of persons with NT1 compared to controls patients with other sleep disorders (OSD) (A**) Distribution of the log-transformed intensity values for all proteins quantified in each LC-MS analysis. Two replicate LC-MS analyses were performed for each of the NT1 patients (n=11) and controls with OSD (n=12), resulting in 45 analytical runs (except for one NT1). Distribution is shown before (left) and after (right) normalization of the data. **(B)** Pearson correlation coefficients for each pairwise comparison between the 46 analytical runs, showing high correlation between the experiments. (**C**) Number of proteins identified by MS/MS sequencing in all the samples and MS experiments (technical MS replicate runs are plotted side by side), indicating homogeneous level of protein identification across samples. (**D**) Data completeness after assignment of protein signals across all runs. Intensity values of proteins that were not sequenced and identified in some of the runs were retrieved by MS signal extraction using the “match between runs” procedure for label-free quantification. The residual number of missing values after this cross-assignment is indicated for each analysis and is typically around 15%. (**E**) Distribution of the coefficients of variation of intensity values across all biological samples, for the entire population of quantified proteins, showing a mode around 25%.

**Supplementary Figure 4**. **Other enriched pathways in NT1 patients. (A)** Log2 fold change of proteins involved in the unfolded protein and response to unfolded proteins in cohort 1 (Top) and cohort 2 (Bottom). **(B)** Log2 fold change of the protein shared between cohort 1 and cohort 2. **(C)** Log2 fold change of proteins involved in the neurotrophin signaling pathway in cohort 2 (Left) and cohort 1 (Right). **(D)** Log2 fold change of neurotrophin signaling pathway proteins shared between cohort 1 and cohort 2. The proteins that are significantly regulated (p-value<0.05; Limma test) in the CSF of NT1 patients are indicated with a star.

**Supplementary Figure 5.** Correlation of mean abundance of the 14 differentially abundant proteins shared between persons with NT1 of both cohorts. Pearson r statistical test was used.

**Supplementary Figure 6.** Boxplots of the shared 14 differentially regulated proteins **(A)** in cohort 1 and **(B)** in cohort 2. P-values are calculated with the Mann-Whitney test. * p-value<0.05, ** p-value <0.01, *** p-value<0.001, **** p-value<0.0001.

**Supplementary figure 7.** Correlation matrix of neurotrophin signaling and complement proteins. The Heatmap shows the r values computed with the Pearson test, highlighted in red are the positive correlations and in blue the negative correlations. Correlations highlighted in yellow are statistically significant.

**Supplementary Figure 8.** Boxplots of the shared 20 complement proteins in recent and long onset disease in NT1 patients. P-values are calculated with the Mann-Whitney test.
