# Supplementary figures and images for: Cerebrospinal fluid proteomics in recent-onset Narcolepsy type 1 reveals activation of the complement system
Source: Front Immunol. 2023 Apr 12;14:1108682. doi: 10.3389/fimmu.2023.1108682 (PMC10130643; doi:10.3389/fimmu.2023.1108682)

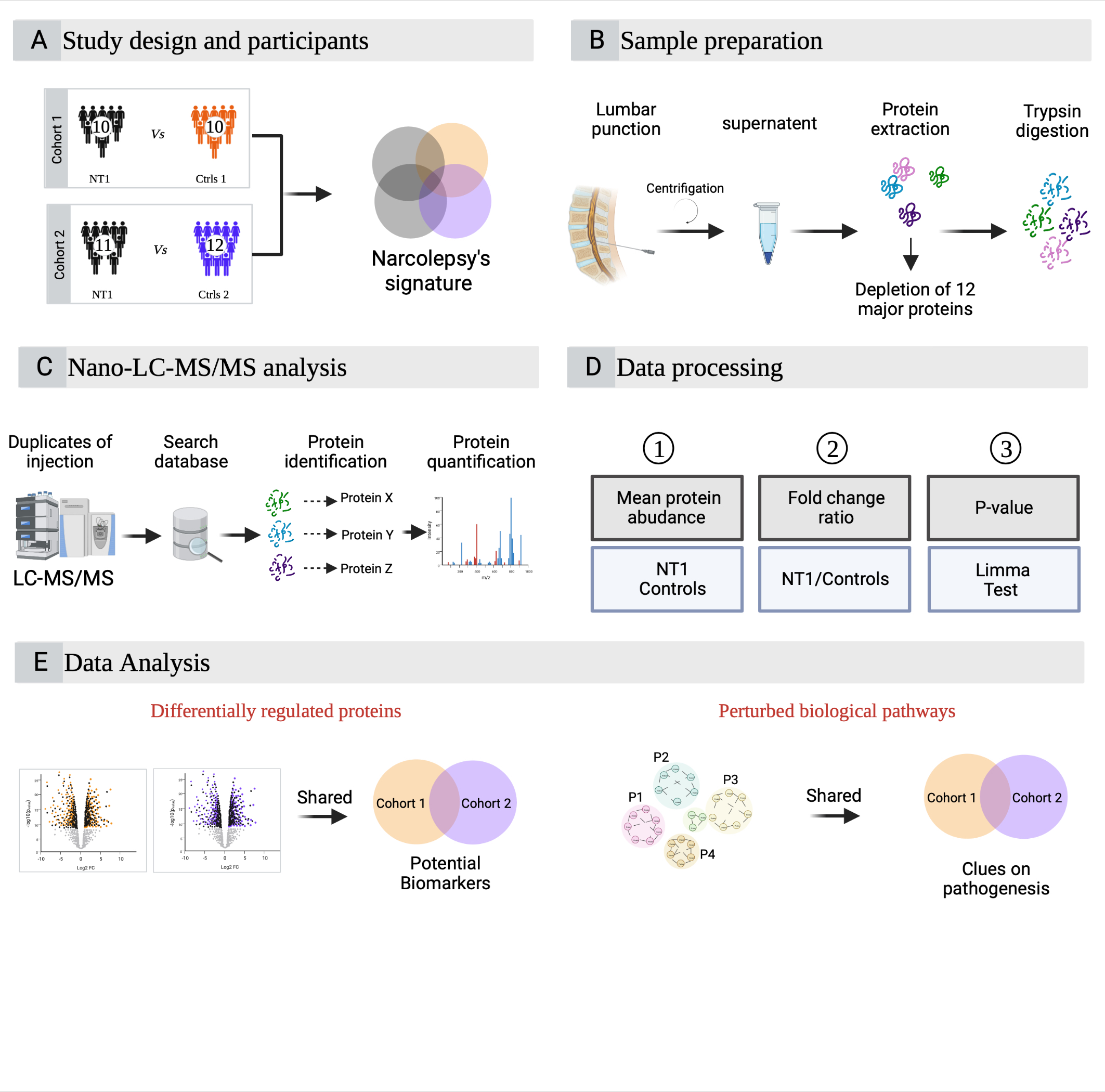

Supplement: Supplementary file 2 [file Image_1.jpeg]

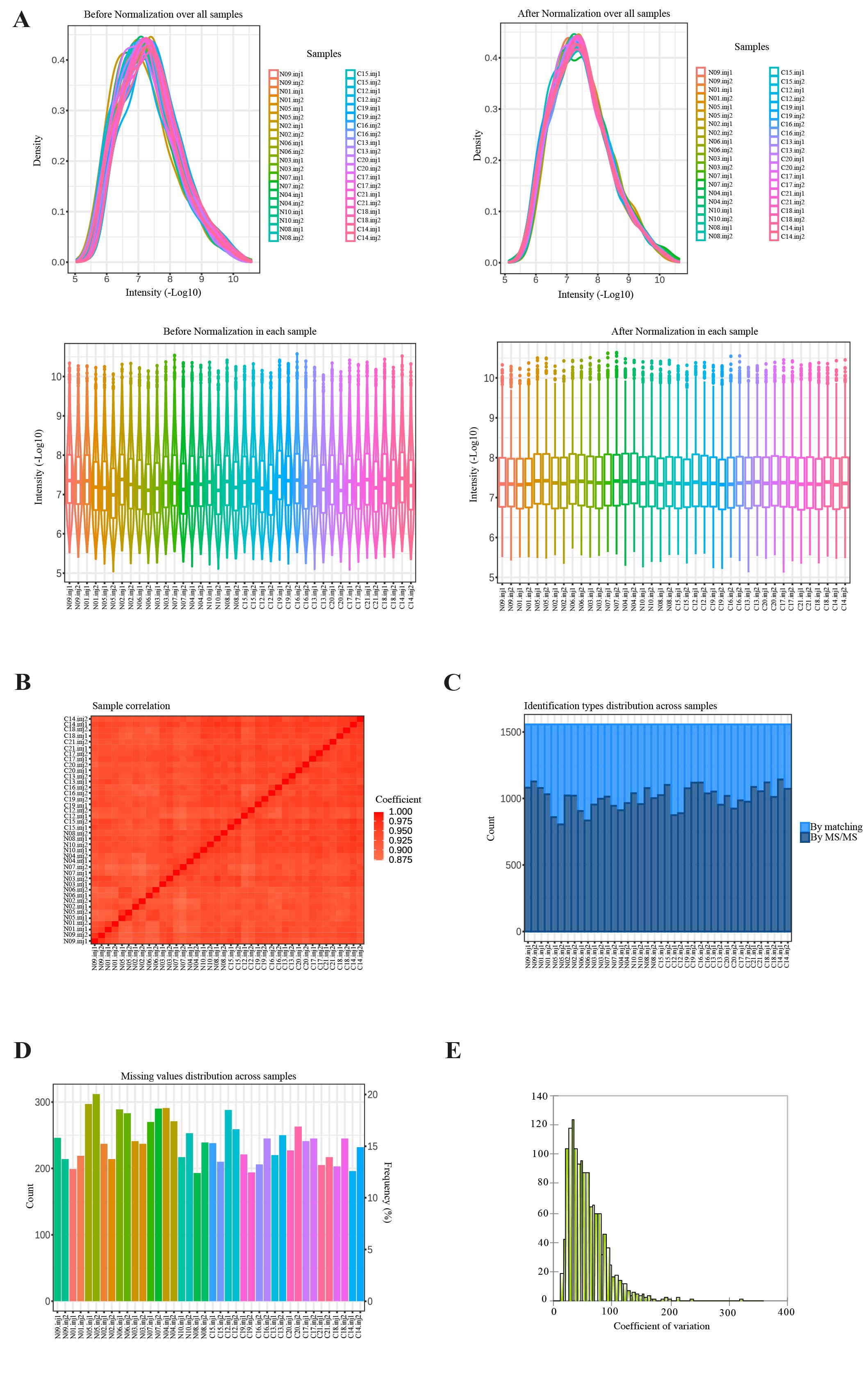

Supplement: Supplementary file 3 [file Image_2.jpeg]

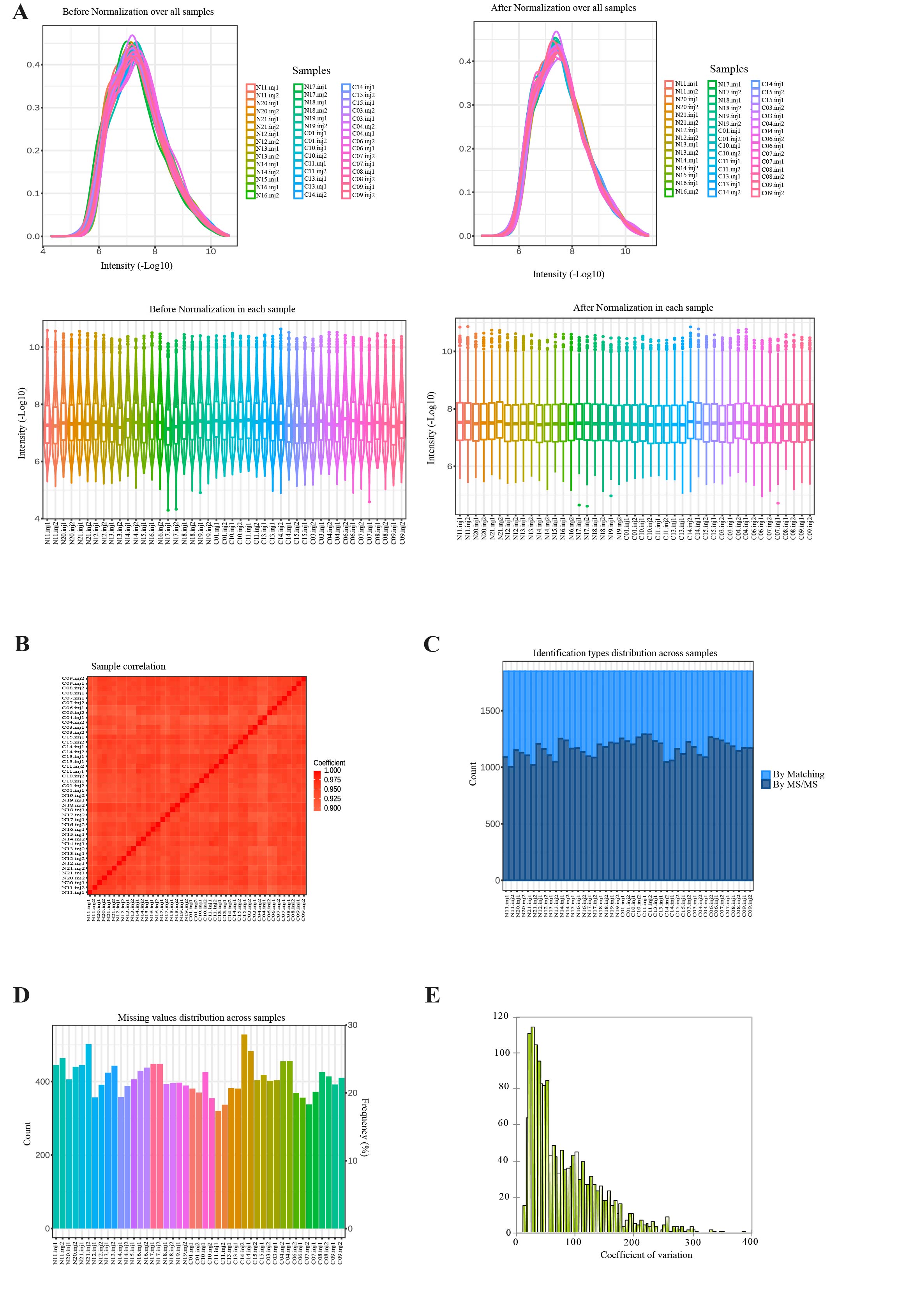

Supplement: Supplementary file 4 [file Image_3.jpeg]

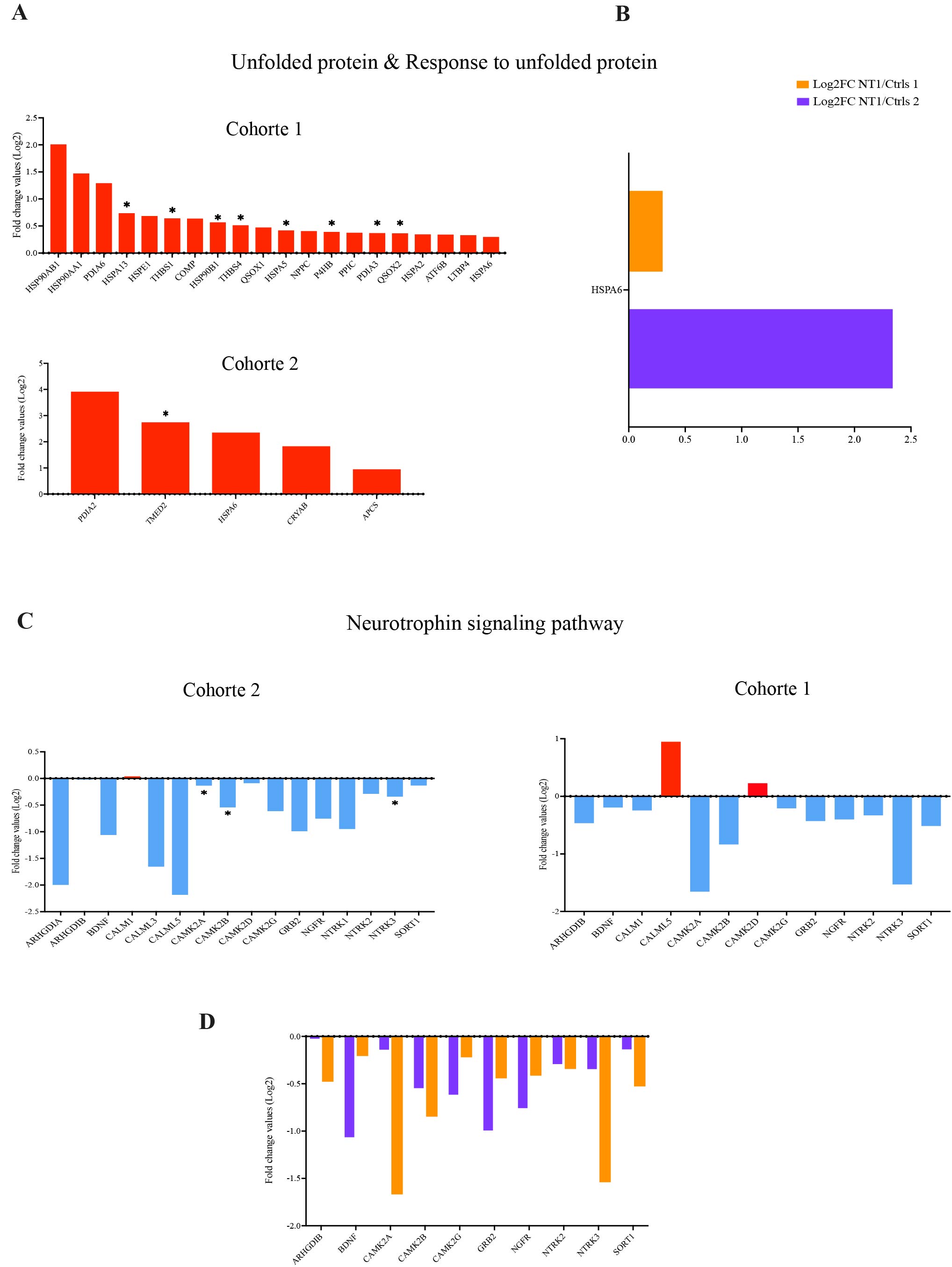

Supplement: Supplementary file 5 [file Image_4.jpeg]

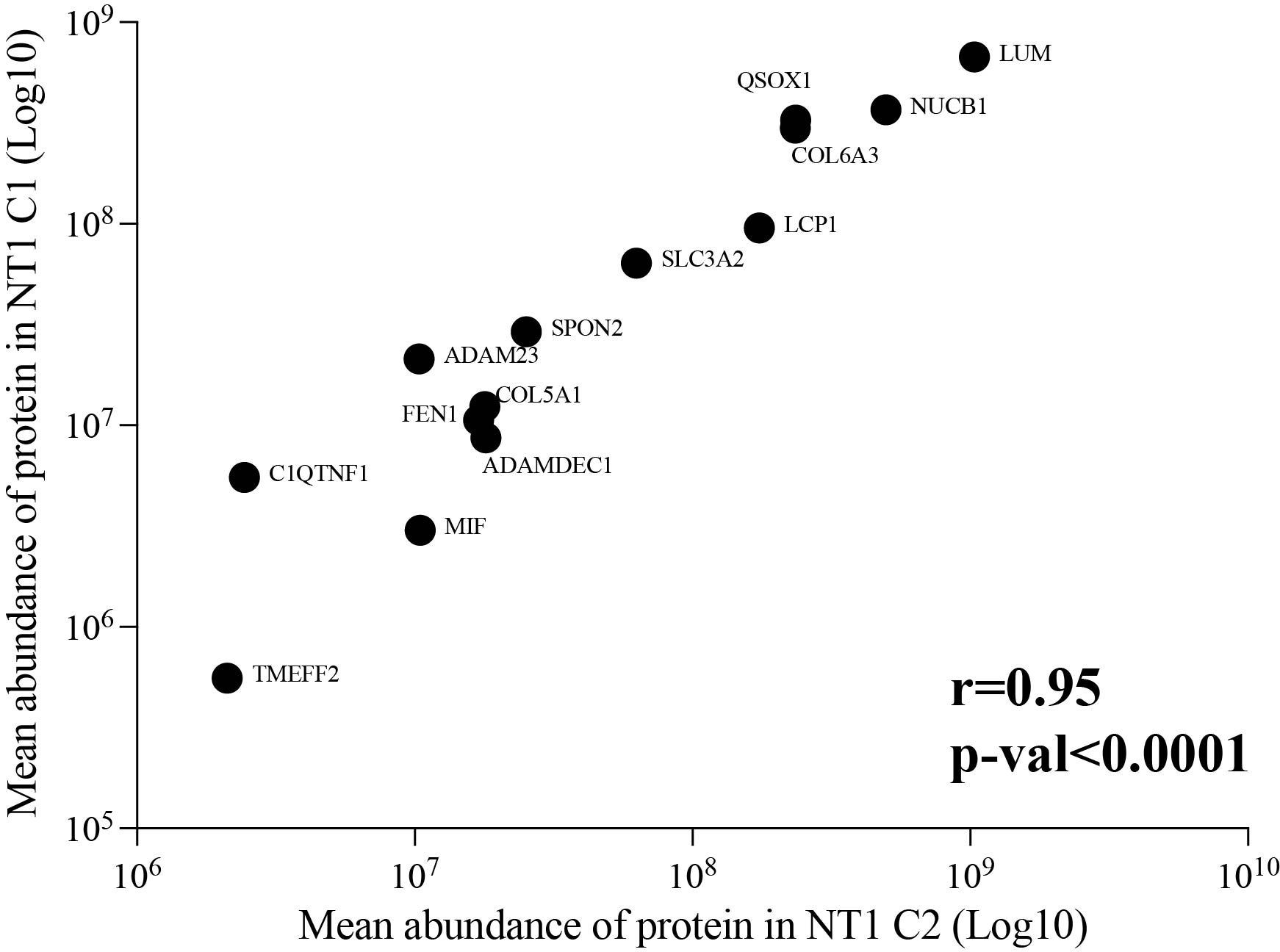

Supplement: Supplementary file 6 [file Image_5.jpeg]

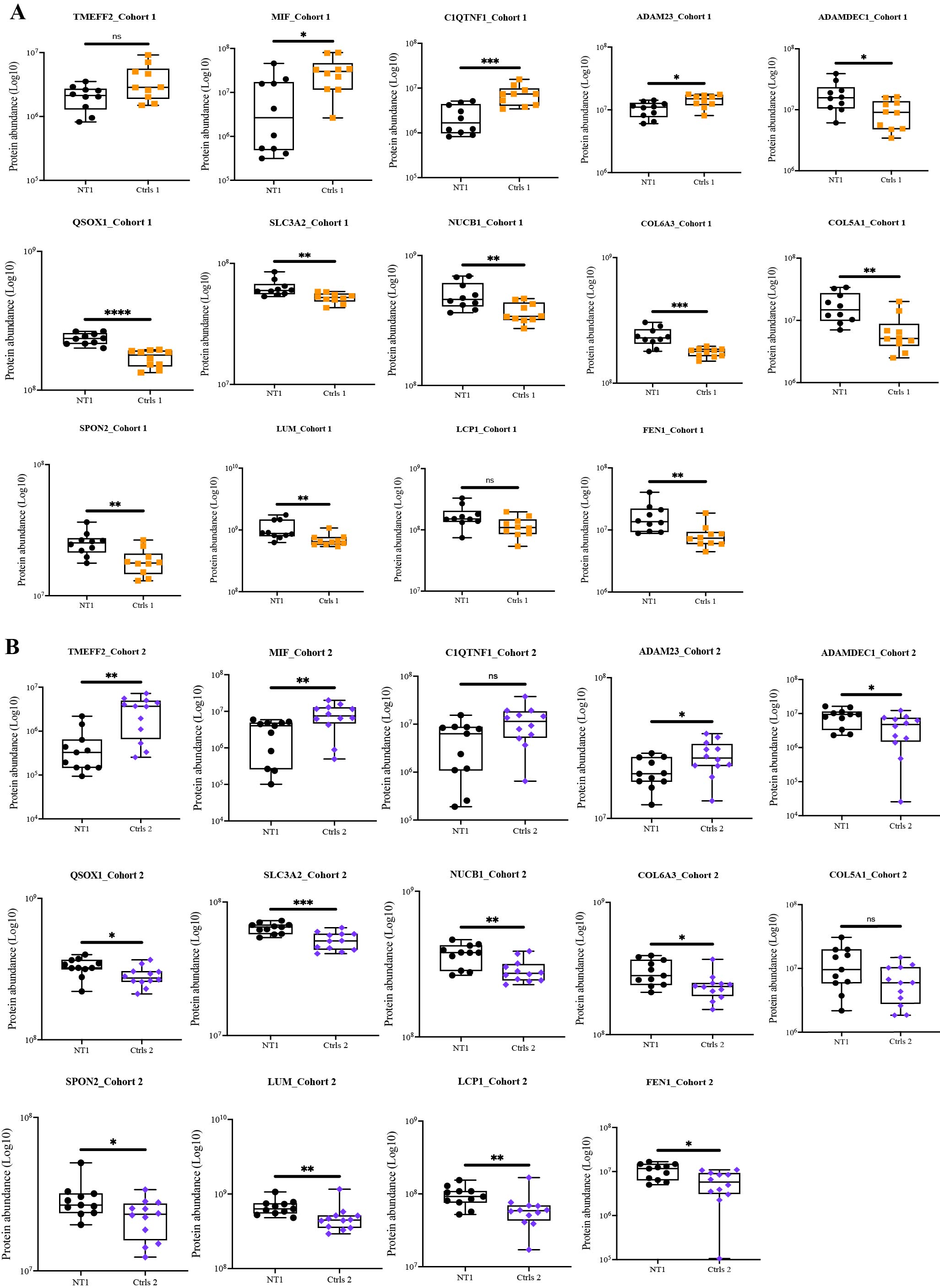

Supplement: Supplementary file 7 [file Image_6.jpeg]

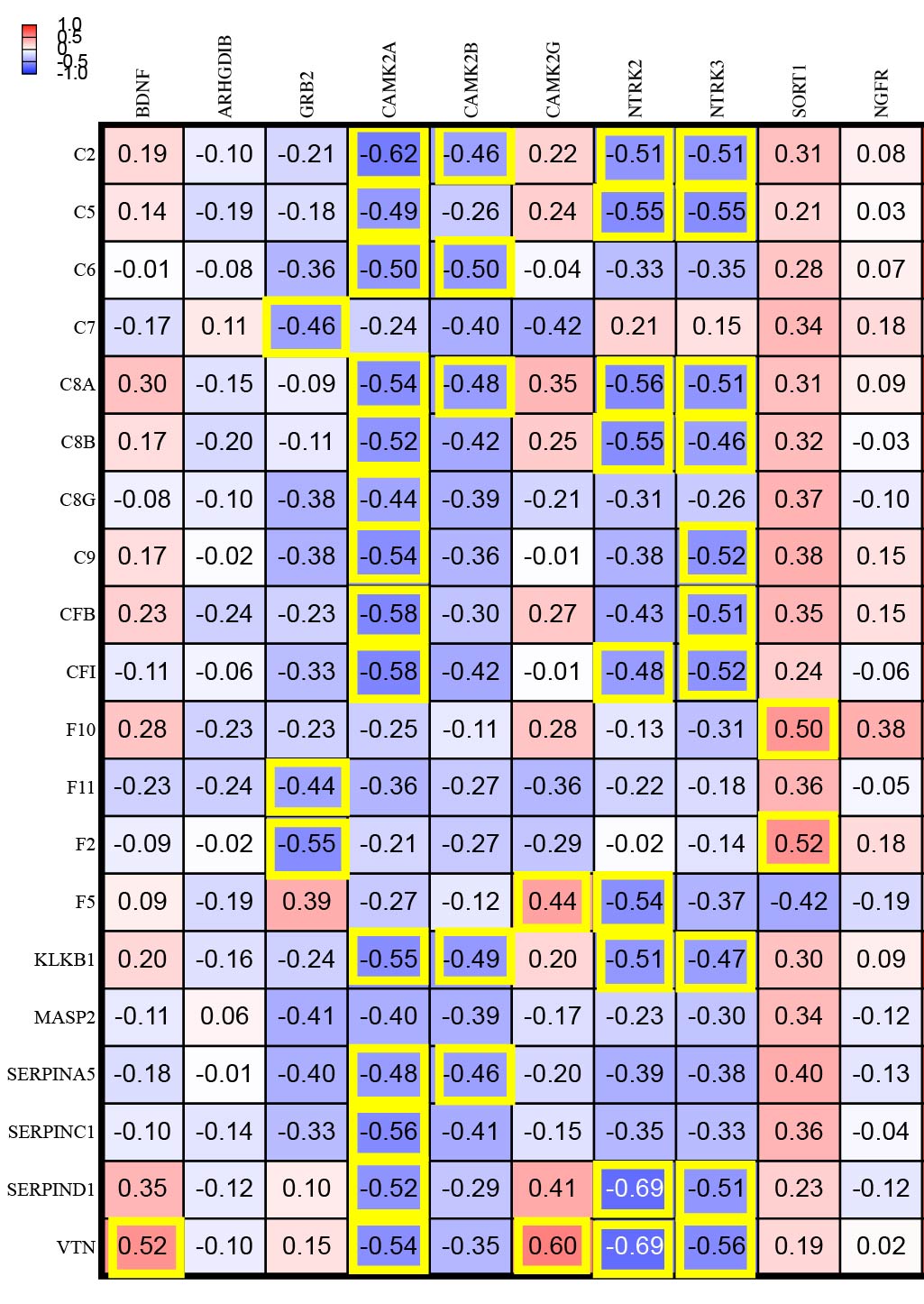

Supplement: Supplementary file 8 [file Image_7.jpeg]

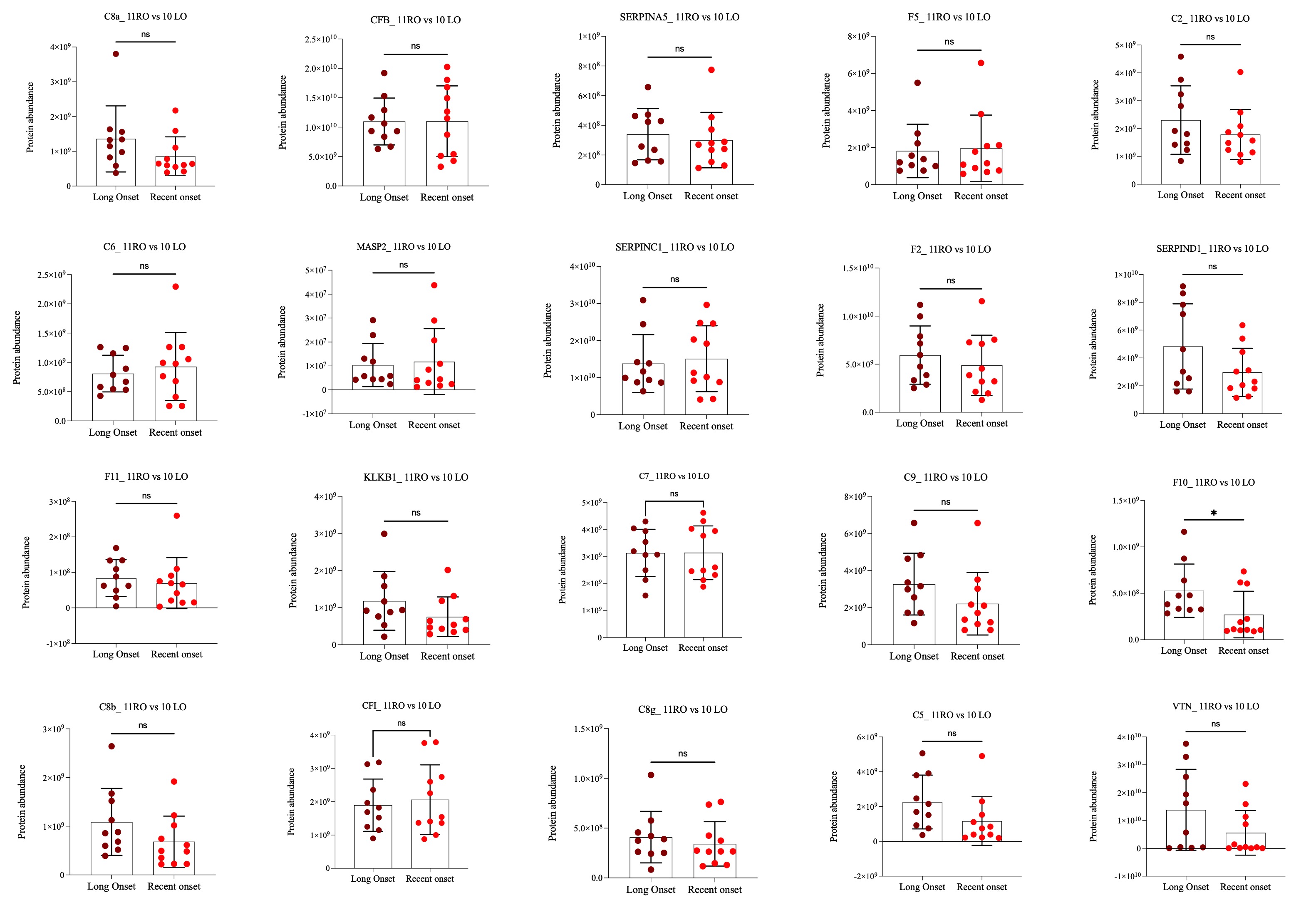

Supplement: Supplementary file 9 [file Image_8.jpeg]
