# Supplementary material for: Cerebrospinal fluid proteomics in recent-onset Narcolepsy type 1 reveals activation of the complement system
Source: Front Immunol. 2023 Apr 12;14:1108682. doi: 10.3389/fimmu.2023.1108682 (PMC10130643; doi:10.3389/fimmu.2023.1108682)
Supplement: Supplementary file 10 [file Table_1.docx]

**Supplementary Table 1. Function of common proteins from “collagen degradation” and “membrane lipid catabolic” pathways. (Source: The Human Protein atlas)**

| **Protein** | **Pathway** | **Function** |
| --- | --- | --- |
| COL5A1 | Collagen degradation pathway | Type V collagen is a member of group I collagen (fibrillar forming collagen). It is a minor connective tissue component of nearly ubiquitous distribution. Type V collagen binds to DNA, heparan sulfate, thrombospondin, heparin, and insulin. |
| COL11A1 |  | May play an important role in fibrillogenesis by controlling lateral growth of collagen II fibrils. |
| COL3A1 |  | Collagen type III occurs in most soft connective tissues along with type I collagen. Involved in regulation of cortical development. Is the major ligand of ADGRG1 in the developing brain and binding to ADGRG1 inhibits neuronal migration and activates the RhoA pathway by coupling ADGRG1 to GNA13 and possibly GNA12. |
| COL6A3 |  | Collagen VI acts as a cell-binding protein. |
| COL1A1 |  | Type I collagen is a member of group I collagen (fibrillar forming collagen). |
| COL1A2 |  | Type I collagen is a member of group I collagen (fibrillar forming collagen). |
| COL6A2 |  | Collagen VI acts as a cell-binding protein. |
| COL14A1 |  | Plays an adhesive role by integrating collagen bundles. It is probably associated with the surface of interstitial collagen fibrils via COL1. The COL2 domain may then serve as a rigid arm which sticks out from the fibril and protrudes the large N-terminal globular domain into the extracellular space, where it might interact with other matrix molecules or cell surface receptors (By similarity). |
| MMP2 |  | Ubiquitous metalloproteinase that is involved in diverse functions such as remodeling of the vasculature, angiogenesis, tissue repair, tumor invasion, inflammation, and atherosclerotic plaque rupture. As well as degrading extracellular matrix proteins, can also act on several nonmatrix proteins such as big endothelial 1 and beta-type CGRP promoting vasoconstriction. Also cleaves KISS at a Gly-\|-Leu bond. Appears to have a role in myocardial cell death pathways. Contributes to myocardial oxidative stress by regulating the activity of GSK3beta. Cleaves GSK3beta in vitro. Involved in the formation of the fibrovascular tissues in association with MMP14 |
| PPT1 | Membrane lipid catabolic pathway | Removes thioester-linked fatty acyl groups such as palmitate from modified cysteine residues in proteins or peptides during lysosomal degradation. Prefers acyl chain lengths of 14 to 18 carbons. |
| HEXA |  | Hydrolyzes the non-reducing end N-acetyl-D-hexosamine and/or sulfated N-acetyl-D-hexosamine of glycoconjugates, such as the oligosaccharide moieties from proteins and neutral glycolipids, or from certain mucopolysaccharides [1,](http://www.ncbi.nlm.nih.gov/pubmed/11707436) [2,](http://www.ncbi.nlm.nih.gov/pubmed/9694901) [3,](http://www.ncbi.nlm.nih.gov/pubmed/8672428) [4](http://www.ncbi.nlm.nih.gov/pubmed/8123671). The isozyme S is as active as the isozyme A on the anionic bis-sulfated glycans, the chondroitin-6-sulfate trisaccharide (C6S-3), and the dermatan sulfate pentasaccharide, and the sulfated glycosphingolipid SM2 [5](http://www.ncbi.nlm.nih.gov/pubmed/11707436). The isozyme B does not hydrolyze each of these substrates, however hydrolyzes efficiently neutral oligosaccharide [6](http://www.ncbi.nlm.nih.gov/pubmed/11707436). Only the isozyme A is responsible for the degradation of GM2 gangliosides in the presence of GM2A |
| SMPDL3B |  | Lipid-modulating phosphodiesterase [1](http://www.ncbi.nlm.nih.gov/pubmed/26095358). Active on the surface of macrophages and dendritic cells and strongly influences macrophage lipid composition and membrane fluidity. Acts as a negative regulator of Toll-like receptor signaling (By similarity). Has in vitro phosphodiesterase activity, but the physiological substrate is unknown [2](http://www.ncbi.nlm.nih.gov/pubmed/26095358). Lacks activity with phosphocholine-containing lipids, but can cleave CDP-choline, and can release phosphate from ATP and ADP (in vitro) (By similarity) |
| HEXB |  | Hydrolyzes the non-reducing end N-acetyl-D-hexosamine and/or sulfated N-acetyl-D-hexosamine of glycoconjugates, such as the oligosaccharide moieties from proteins and neutral glycolipids, or from certain mucopolysaccharides [1,](http://www.ncbi.nlm.nih.gov/pubmed/11707436) [2,](http://www.ncbi.nlm.nih.gov/pubmed/9694901) [3,](http://www.ncbi.nlm.nih.gov/pubmed/8672428) [4](http://www.ncbi.nlm.nih.gov/pubmed/8123671). The isozyme B does not hydrolyze each of these substrates, however hydrolyzes efficiently neutral oligosaccharide [5](http://www.ncbi.nlm.nih.gov/pubmed/11707436). Only the isozyme A is responsible for the degradation of GM2 gangliosides in the presence of GM2A [6,](http://www.ncbi.nlm.nih.gov/pubmed/9694901) [7,](http://www.ncbi.nlm.nih.gov/pubmed/8672428) [8](http://www.ncbi.nlm.nih.gov/pubmed/8123671). During fertilization is responsible, at least in part, for the zona block to polyspermy. Present in the cortical granules of non-activated oocytes, is exocytosed during the cortical reaction in response to oocyte activation and inactivates the sperm galactosyltransferase-binding site, accounting for the block in sperm binding to the zona pellucida (By similarity) |
| CEL |  | Catalyzes the hydrolysis of a wide range of substrates including cholesteryl esters, phospholipids, lysophospholipids, di- and tri-acylglycerols, and fatty acid esters of hydroxy fatty acids (FAHFAs) [1,](http://www.ncbi.nlm.nih.gov/pubmed/8471055) [2,](http://www.ncbi.nlm.nih.gov/pubmed/27509211) [3,](http://www.ncbi.nlm.nih.gov/pubmed/10220579) [4](http://www.ncbi.nlm.nih.gov/pubmed/27650499). Preferentially hydrolyzes FAHFAs with the ester bond further away from the carboxylate. Unsaturated FAHFAs are hydrolyzed more quickly than saturated FAHFAs (By similarity). Has an essential role in the complete digestion of dietary lipids and their intestinal absorption, along with the absorption of fat-soluble vitamins |
| ASAH1 |  | Lysosomal ceramidase that hydrolyzes sphingolipid ceramides into sphingosine and free fatty acids at acidic pH [1,](http://www.ncbi.nlm.nih.gov/pubmed/10610716) [2,](http://www.ncbi.nlm.nih.gov/pubmed/7744740) [3,](http://www.ncbi.nlm.nih.gov/pubmed/15655246) [4](http://www.ncbi.nlm.nih.gov/pubmed/11451951). Ceramides, sphingosine, and its phosphorylated form sphingosine-1-phosphate are bioactive lipids that mediate cellular signaling pathways regulating several biological processes including cell proliferation, apoptosis and differentiation [5](http://www.ncbi.nlm.nih.gov/pubmed/10610716). Has a higher catalytic efficiency towards C12-ceramides versus other ceramides [6,](http://www.ncbi.nlm.nih.gov/pubmed/7744740) [7](http://www.ncbi.nlm.nih.gov/pubmed/15655246). Also catalyzes the reverse reaction allowing the synthesis of ceramides from fatty acids and sphingosine [8,](http://www.ncbi.nlm.nih.gov/pubmed/12764132) [9](http://www.ncbi.nlm.nih.gov/pubmed/12815059). For the reverse synthetic reaction, the natural sphingosine D-erythro isomer is more efficiently utilized as a substrate compared to D-erythro-dihydrosphingosine and D-erythro-phytosphingosine, while the fatty acids with chain lengths of 12 or 14 carbons are the most efficiently used [10](http://www.ncbi.nlm.nih.gov/pubmed/12764132). Has also an N-acylethanolamine hydrolase activity [11](http://www.ncbi.nlm.nih.gov/pubmed/15655246). By regulating the levels of ceramides, sphingosine and sphingosine-1-phosphate in the epidermis, mediates the calcium-induced differentiation of epidermal keratinocytes [12](http://www.ncbi.nlm.nih.gov/pubmed/17713573). Also indirectly regulates tumor necrosis factor/TNF-induced apoptosis (By similarity). By regulating the intracellular balance between ceramides and sphingosine, in adrenocortical cells, probably also acts as a regulator of steroidogenesis |
