# Supplementary material for: Cerebrospinal fluid proteomics in recent-onset Narcolepsy type 1 reveals activation of the complement system
Source: Front Immunol. 2023 Apr 12;14:1108682. doi: 10.3389/fimmu.2023.1108682 (PMC10130643; doi:10.3389/fimmu.2023.1108682)
Supplement: Supplementary file 11 [file Table_2.docx]

***Supplementary Table 2. Function of the 14 shared proteins between cohorts.***

| Protein | Main function | Reference |
| --- | --- | --- |
| **TMEFF2** | Transmembrane protein with growth factor (EGF)-like and two follistatin like domains 2 is a putative transmembrane protein. It is predominantly expressed in the brain and promotes the survival of hippocampal and mesencephalic neurons. | (8) |
| **MIF** | Macrophage migration inhibitory factor is a pleiotropic cytokine produced by several cell types of the innate and adaptive immune system and is widely expressed in neural tissues. The role of MIF has been studied in various inflammatory and neurodegenerative disease. Results show either a protective or a pathogenic role depending on the disease. | (19),(20) |
| **C1QTNF1** | C1q/TNF-related protein1 is a secreted protein mostly expressed in adipose tissues with major functions in glucolipid metabolism, inflammation, cell proliferation, and apoptosis. | (21) |
| **ADAM23** | ADAM Metallopeptidase Domain 23 is a cell surface glycoprotein whose expression is confined to nervous tissue, specifically to neurons. It has a role in cell-cell interactions. | (22) |
| **QSOX1** | Quiescin sulfhydryl oxidase 1 catalyzes the oxidation of sulfhydryl groups in peptide and protein thiols to disulfides with the reduction of oxygen to hydrogen peroxide. It plays a role in disulfide bond formation in a variety of extracellular proteins. | (23) |
| **SLC3A2** | solute carrier family 3 member 2 is highly expressed on proliferating lymphocytes and other rapidly growing cells. It mediates integrin dependent cell spreading, cell migration and protection from apoptosis. | (24) |
| **NUCB1** | Nucleobindin 1 is a putative DNA- and calcium-binding protein. NUCB1 is a pan neuronal marker in all brain regions and the spinal cord and is co-expressed with NUCB2 in a subset of hypothalamic neurons. | (25) |
| **COL6A3** | Collagen VI is a major extracellular matrix protein that acts as a regulator of Schwann cell differentiation and has a role in preserving peripheral nerve myelination, function and structure. It has also a function of orchestrating nerve regeneration after injury. | (26) |
| **SPON2** | Spondin 2 is a cell adhesion protein that promotes adhesion and outgrowth of hippocampal embryonic neurons. Binds directly to bacteria and their components and functions as an opsonin for macrophage phagocytosis of bacteria. Essential in the initiation of the innate immune response and represents a unique pattern-recognition molecule in the ECM for microbial pathogens. | Uniprot |
| **LUM** | Luminican is a member of the small leucine-rich proteoglycan (SLRP) family that includes decorin, biglycan, fibromodulin, keratocan, epiphycan, and osteoglycin. In these bifunctional molecules, the protein moiety binds collagen fibrils and the highly charged hydrophilic glycosaminoglycans regulate interfibrillar spacings. Lumican is the major keratan sulfate proteoglycan of the cornea but is also distributed in interstitial collagenous matrices throughout the body. Lumican may regulate collagen fibril organization and circumferential growth, corneal transparency, and epithelial cell migration and tissue repair. [provided by RefSeq, Jul 2008] | Uniprot |
| **LCP1** | Lymphocyte cytosolic protein 1 actin-binding protein plays a role in the activation of T cells in response to costimulation through TCR/CD3 and CD2 or CD28. Modulates the cell surface expression of IL2RA/CD25 and CD69. | (Uniprot) |
| **FEN1** | Structure-specific nuclease with 5'-flap endonuclease and 5'-3' exonuclease activities involved in DNA replication and repair. During DNA replication, cleaves the 5'-overhanging flap structure that is generated by displacement synthesis when DNA polymerase encounters the 5'-end of a downstream Okazaki fragment. Also involved in the long patch base excision repair (LP-BER) pathway, by cleaving within the apurinic/apyrimidinic (AP) site-terminated flap. Acts as a genome stabilization factor that prevents flaps from equilibrating into structures that lead to duplications and deletions. Also possesses 5'-3' exonuclease activity on nicked or gapped double-stranded DNA, and exhibits RNase H activity. FEN1 is also involved in replication and repair of rDNA and in repairing mitochondrial DNA. | Uniprot |
| **ADAMDEC1** | A disintegrin and metalloproteinase domain-like protein decysin-1. May play an important role in the control of the immune response and during pregnancy. Its expression is up-regulated during dendritic cells maturation. This protein may play an important role in dendritic cell function and their interactions with germinal center T cells. [provided by RefSeq, Jul 2008] | (Uniprot) |
| **COL5A1** | Collagen alpha-1(V) chain Type V collagen is a member of group I collagen (fibrillar forming collagen). It is a minor connective tissue component of nearly ubiquitous distribution. Type V collagen binds to DNA, heparan sulfate, thrombospondin, heparin, and insulin. | (Uniprot) |
